# Dapagliflozin Protects Cardiomyocytes against Doxorubicin-Induced Toxicity by Modulating Sirtuin 1/Sirtuin 3 and Ferroptosis Pathway

**DOI:** 10.1021/acsptsci.5c00760

**Published:** 2026-04-03

**Authors:** Hnin Ei Ei Khine, Supachoke Mangmool, Warisara Parichatikanond

**Affiliations:** † Department of Pharmacology, Faculty of Pharmacy, 68022 Mahidol University, Bangkok 10400, Thailand; ‡ Department of Pharmaceutical Care, Faculty of Pharmacy, 68021 Chiang Mai University, Chiang Mai 50200, Thailand; § Centre of Biopharmaceutical Science for Healthy Ageing, Faculty of Pharmacy, Mahidol University, Bangkok 10400, Thailand; ∥ Centre of Molecular Targeting and Integrated Drug Development, Faculty of Pharmacy, Mahidol University, Bangkok 10400, Thailand

**Keywords:** cardioprotection, dapagliflozin, doxorubicin, SIRT1/SIRT3, ferroptosis, mitochondrial dysfunction

## Abstract

Cardiac dysfunction can be aggravated by chemotherapeutic agents, including doxorubicin, through mechanisms involving mitochondrial dysfunction, elevated oxidative stress, suppression of sirtuin (SIRT1/SIRT3) signaling, and activation of apoptotic and ferroptotic pathways. Dapagliflozin, a selective sodium-glucose cotransporter 2 (SGLT2) inhibitor, has been demonstrated to possess cardioprotective effects; however, the interplay between sirtuin signaling and ferroptosis in dapagliflozin-mediated cardioprotection under doxorubicin-induced stress remains unclear. In the present study, dapagliflozin restored cellular function in H9c2 cardiomyoblasts exposed to doxorubicin by reducing apoptosis, oxidative stress, and lipid peroxidation, while preserving mitochondrial respiration and glycolytic function. Dapagliflozin reversed doxorubicin-induced downregulation of SIRT1, SIRT3, GPX4, BCL2, OPA1, and PGC1α, and mitigated the upregulation of ACSL4, BAX, and DNM1 at both transcriptional and translational levels. The cardioprotective efficacy of dapagliflozin under cellular stress depends critically on SIRT1/SIRT3 signaling and ferroptosis regulation, as pharmacological inhibition of these sirtuins abolished its protective potentials; conversely, these effects were enhanced by ferroptosis suppression and attenuated by its induction. Furthermore, dapagliflozin-mediated inhibition of ferroptosis downregulated SIRT1/SIRT3 expression, suggesting a potential feedback mechanism under chemotherapeutic stress. Notably, sirtuin inhibition compromised these protective responses despite ferroptosis blockade, highlighting SIRT1/SIRT3 as upstream regulators of dapagliflozin-mediated cardioprotection and underscoring the necessity of sirtuin activity for ferroptosis suppression. Collectively, these findings reveal that dapagliflozin mitigates doxorubicin-induced cardiotoxicity via the coordinated regulation of SIRT1/SIRT3 signaling and ferroptosis pathways, involving key mediators of apoptosis, mitochondrial dynamics, and lipid metabolism.

## Graphical abstract

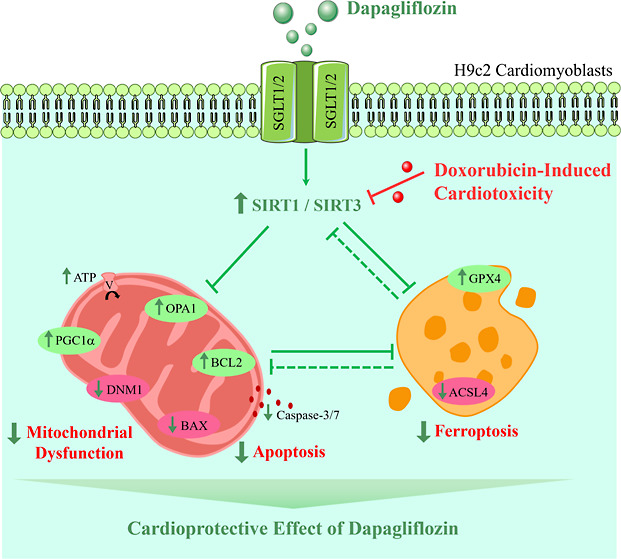

1

Heart failure reflects a progressive decline in cardiomyocyte function and myocardial integrity, resulting in ventricular dysfunction, arrhythmias, and eventual decompensation.^1^ Despite diverse etiologies, including genetic, metabolic, and cardiotoxic drug exposure, heart failure shares common pathological mechanisms involving mitochondrial dysfunction, oxidative stress, and regulated cell death pathways, particularly apoptosis and ferroptosis.^2,3^ Failing hearts exhibit impaired energy metabolism, mitochondrial disorganization, and oxidative damage, leading to cardiomyocyte loss.^4^ Mitochondria are central to these processes, controlling adenosine triphosphate (ATP) synthesis as well as reactive oxygen species (ROS) levels, calcium homeostasis, and cell death pathways.^4^ Mitochondrial dynamics rely on a balance between optic atrophy 1 (OPA1)-mediated fusion and dynamin-related protein 1 (DRP1)-driven fission, with stress-induced DRP1 upregulation and OPA1 suppression promoting fragmentation, ROS accumulation, and energy failure.^5^ Furthermore, chronic cardiac stress downregulates peroxisome proliferator-activated receptor gamma coactivator 1-alpha (PGC-1α), impairing mitochondrial biogenesis, ATP availability, and antioxidant capacity, thus worsening bioenergetic decline.^6,7^

Significant attention has focused on sirtuins, a family of nicotinamide adenine dinucleotide (NAD^+^)-dependent deacetylases that play a crucial role in stress adaptation. Among them, sirtuin 1 (SIRT1) and sirtuin 3 (SIRT3) are key regulators of mitochondrial function, oxidative stress defenses, and cell death pathways.^8^ SIRT1, predominantly localized in the nucleus, facilitates mitochondrial biogenesis via PGC-1α activation^8^ and suppresses apoptosis by repressing p53-dependent transcription of proapoptotic markers such as B-cell lymphoma 2-associated X protein (BAX).^9^ SIRT3, localized within the mitochondria, supports ATP synthesis and reduces ROS generation through the deacetylation of mitochondrial superoxide dismutase 2 (SOD2),^10,11^ while preserving mitochondrial dynamics by enhancing OPA1 and limiting fission.^12^ Both sirtuins modulate ferroptosis, with SIRT1 helping to maintain antioxidant defenses that support glutathione peroxidase 4 (GPX4) activity^13^ and SIRT3 reducing lipid peroxidation and mitochondrial injury.^14^ Together, the SIRT1/SIRT3 axis forms a central regulatory mechanism that promotes cardiomyocyte survival.

Ferroptosis, a regulated form of cell death characterized by iron-dependent lipid peroxidation, has emerged as a significant contributor to cardiac injury. Unlike apoptosis, ferroptosis involves catastrophic membrane failure driven by ROS accumulation.^15^ GPX4 suppresses ferroptosis by detoxifying lipid hydroperoxides,^16^ while acyl-coenzyme A synthetase long-chain family member 4 (ACSL4) promotes it by enhancing membrane lipid oxidation susceptibility.^17^ In models of heart failure and cardiotoxicity, decreased GPX4 and increased ACSL4 levels have been observed, implicating ferroptosis as a major mechanism of cardiomyocyte loss.^16,17^ Importantly, ferroptosis intersects with apoptosis, which is mediated by BAX-induced mitochondrial outer membrane permeabilization, resulting in cytochrome c release and caspase activation. In failing hearts, BAX is upregulated, and the antiapoptotic protein B-cell lymphoma 2 (BCL2) is suppressed, reflecting a shift toward proapoptotic signaling.^18^

Doxorubicin, a widely used anthracycline chemotherapy drug, is constrained by cumulative and irreversible cardiac toxicity, exemplifying the mechanisms underlying such cardiotoxic potentials. It generates ROS via redox cycling with iron, disrupts mitochondrial respiration, damages mitochondrial DNA, and depletes ATP levels.^19^ These events activate apoptosis through the tumor suppressor protein p53 and BAX signaling,^20^ and trigger ferroptosis via GPX4 depletion and iron overload.^21^ Doxorubicin also suppresses sirtuin activity, whereby SIRT1 mitigates oxidative stress and inflammation by repressing p38 mitogen-activated protein kinase (MAPK) signaling,^22^ while SIRT3 counters hypertrophy and mitochondrial dysfunction through antioxidant mechanisms and BCL2-interacting protein 3 (BNIP3)-mediated pathways.^23^ However, how sirtuin suppression is linked to ferroptosis in doxorubicin-triggered cardiac injury remains poorly defined.

Dapagliflozin, primarily a sodium-glucose cotransporter 2 (SGLT2) inhibitor with slight activity at SGLT1, has shown notable cardioprotective responses independent of its glucose-lowering properties.^24,25^ Landmark clinical trials have demonstrated that dapagliflozin lowers the incidence of heart failure events and cardiovascular mortality in both diabetic and nondiabetic patients.^26,27^ Mechanistic studies suggest that dapagliflozin enhances mitochondrial function, restores ATP production, and reduces oxidative stress via upregulation of SIRT1 and SIRT3 in various cell types, including skeletal muscle cells and cardiomyocytes.^28,29^ Dapagliflozin not only protects against ferroptosis by preserving GPX4 and limiting lipid peroxidation in cardiomyopathy models,^30^ but also promotes mitochondrial integrity via enhanced fusion and biogenesis, and activates PGC-1α signaling.^31^ Additionally, dapagliflozin restores the BCL2/BAX ratio and alleviates endoplasmic reticulum stress, thereby protecting doxorubicin-treated cardiomyocytes against apoptosis.^32^

Although dapagliflozin targets multiple critical pathways, its effects have not been fully explored within the integrated framework of cardiotoxicity, where mitochondrial dysfunction, ferroptosis, and sirtuin signaling intersect. This study examines whether dapagliflozin protects cardiomyocytes against doxorubicin-induced cellular injury by activating the SIRT1/SIRT3 axis, suppressing apoptosis and ferroptosis, regulating mitochondrial function, and mitigating oxidative stress, with the goal of providing new insights into its cardioprotective properties and potential to attenuate chemotherapy-related cardiac injury.

## 2 Materials and Methods

### 2.1 Materials

Doxorubicin hydrochloride, dapagliflozin, EX-527 (a SIRT1 inhibitor), 3-TYP (a SIRT3 inhibitor), ferrostatin-1 (Fer-1; a ferroptosis inhibitor), and erastin (a ferroptosis inducer) were purchased from MedChemExpress (Monmouth Junction, NJ, USA). Cell culture reagents, including Dulbecco’s Modified Eagle Medium (DMEM), fetal bovine serum (FBS), penicillin-streptomycin-amphotericin B (P/S/A), and 0.25% trypsin–EDTA, were obtained from Gibco (Grand Island, NY, USA). Additional reagents such as 3-(4,5-dimethylthiazol-2-yl)-2,5-diphenyl tetrazolium bromide (MTT), dimethyl sulfoxide (DMSO), and skim milk powder were sourced from Sigma-Aldrich (St. Louis, MO, USA).

### 2.2 Cell Culture

The H9c2 rat cardiomyoblasts (American Type Culture Collection; ATCC, CRL-1466; Manassas, VA, USA) were cultured in DMEM supplemented with 10% FBS and 1% P/S/A. Cells were maintained at 37 °C in a 5% CO_2_ humidified incubator and subcultured using 0.25% trypsin–EDTA once they reached approximately 80% confluency to support consistent proliferation.

### 2.3 Cell Viability Assay

Cell viability was determined using the MTT colorimetric assay.^33^ H9c2 cells were seeded in 96-well plates at a density of 1 × 10^4^ cells per well and incubated overnight. Cells were then pretreated with EX-527 (1 μM), 3-TYP (5 μM), Fer-1 (1 μM), or erastin (3 μM) for 1 h. After pretreatment, dapagliflozin (10 μM) was added for 6 h, followed by exposure to doxorubicin (0.1 μM) for 24 h. The culture medium was then replaced with MTT solution (0.5 mg/mL), and cells were incubated in the dark for 3 h. Formazan crystals formed were dissolved in DMSO, and absorbance was measured at 570 nm using a Synergy HTX microplate reader (BioTek, Winooski, VT, USA). Cell viability was expressed as a percentage relative to untreated controls.

### 2.4 Caspase-3/7 Activity Assay

Apoptotic activity in H9c2 cells was evaluated using the Caspase-Glo 3/7 assay kit (Promega, Madison, WI, USA).^34^ Cells were plated in 96-well plates at a density of 1 × 10^4^ cells per well and allowed to incubate overnight. After treatments, 100 μL of Caspase-Glo 3/7 reagent was added to each well, followed by incubation in the dark for 30 min with gentle shaking. Luminescence was measured using a Synergy HTX microplate reader (λ_Em_ = 528 nm). Results were normalized to the untreated control group and expressed as a percentage.

### 2.5 Annexin V/Propidium Iodide (PI) Apoptosis Analysis

Apoptotic and necrotic cell populations were quantified using Annexin V-FITC/PI staining (Sigma-Aldrich) according to the manufacturer’s instructions. H9c2 cells were seeded in 6-well plates at a density of 2 × 10^6^ cells per well and incubated overnight. Following treatment, cells were harvested by trypsinization, washed twice with phosphate-buffered saline (PBS), and resuspended in 1× binding buffer at a concentration of 1 × 10^6^ cells/mL. A 300 μL volume of this suspension was incubated with 5 μL Annexin V-FITC and 10 μL PI for 30 min at room temperature in the dark. Stained cells were then analyzed using a CytoFLEX-S flow cytometer (Beckman Coulter, Brea, CA, USA), and results were processed with FlowJo V10 software (BD, Franklin Lakes, NJ, USA). Cell populations were categorized as viable (Q1: Annexin V^–^/PI^–^), early apoptotic (Q2: Annexin V^+^/PI^–^), late apoptotic (Q3: Annexin V^+^/PI^+^), or necrotic (Q4: Annexin V^–^/PI^+^). Data were presented as the percentage of each population relative to untreated controls.

### 2.6 Ferroptosis Assessment

Lipid peroxidation, a hallmark of ferroptosis, was quantified using the oxidation-sensitive fluorescent probe C11-BODIPY (581/591) (Thermo Fisher Scientific, Waltham, MA, USA) as per the manufacturer’s protocol. H9c2 cells were seeded in 6-well plates at a density of 2 × 10^6^ cells per well and incubated overnight. Following treatment, cells were harvested by trypsinization, washed with cold PBS, and incubated with 2 μM C11-BODIPY (581/591) at 37 °C in the dark for 30 min. After incubation, cells were washed again with cold PBS. Fluorescence was measured using a CytoFLEX-S flow cytometer, with excitation at 488 nm and emission collected in the red (585/42 nm) and green (525/40 nm) channels. Data were analyzed using FlowJo V10 software. An increase in green fluorescence and a decrease in red fluorescence indicate elevated lipid peroxidation associated with ferroptosis. Results were expressed as the percentage of oxidized C11-BODIPY-positive cells relative to untreated controls.

### 2.7 Reactive Oxygen Species Assessment

Mitochondrial ROS levels were evaluated using MitoSOX Red (Invitrogen, Waltham, MA, USA).^33^ H9c2 cells were seeded overnight on 2% gelatin-coated coverslips in 12-well plates at a density of 1 × 10^5^ cells per well. Following treatments, cells were washed with PBS and incubated with 2.5 μM MitoSOX Red at 37 °C in the dark for 30 min. After washing with PBS, coverslips were mounted with ProLong Diamond Antifade Mountant (Invitrogen), and fluorescence images were acquired at 20× magnification (λ_Ex_/λ_Em_: 396/610 nm) using a Nikon Eclipse Ti2E inverted fluorescence microscope (Nikon Instruments, Melville, NY, USA). Fluorescence intensity was quantified using ImageJ (Version 1.8.0–172; NIH, Bethesda, MD, USA) by analyzing at least 100 randomly selected cells per group.

Intracellular ROS generation was measured using 2′,7′-dichlorodihydrofluorescein diacetate (DCFH-DA) (Sigma-Aldrich).^33^ H9c2 cells were seeded overnight in 6-well plates at a density of 2 × 10^6^ cells per well. After treatments, cells were washed with PBS and incubated with 10 μM DCFH-DA at 37 °C in the dark for 30 min. Fluorescence signals were quantified using a CytoFLEX-S flow cytometer and analyzed with FlowJo V10 software. Results were expressed as a percentage relative to untreated controls.

### 2.8 Mitochondrial Respiration Determination

Mitochondrial respiration in live H9c2 cells was assessed using the Seahorse XF Cell Mito Stress Test Kit (Agilent Technologies, Santa Clara, CA, USA) on the Seahorse XF96 Analyzer (Agilent Technologies).^34^ Cells were seeded in XF96-well microplates (Agilent Technologies) at a density of 1.5 × 10^4^ cells per well and incubated overnight. After treatment, the growth medium was replaced with prewarmed Seahorse XF Base Medium (Agilent Technologies) supplemented with 1 mM pyruvate, 2 mM l-glutamine, and 10 mM glucose (pH 7.4) at 180 μL per well. Oxygen consumption rate (OCR) was measured under basal conditions and sequentially after injections of 1 μM oligomycin (an ATP synthase inhibitor), 2 μM FCCP (an oxidative phosphorylation uncoupler), and a combination of 0.5 μM rotenone and 0.5 μM antimycin A (mitochondrial complex I and III inhibitors). OCR data were analyzed to determine bioenergetic parameters, including basal respiration, ATP-linked respiration, maximal respiration, and spare respiratory capacity.

### 2.9 Mitochondrial Glycolytic Activity Assay

The glycolytic profile of live H9c2 cells was evaluated with the Seahorse XF Glycolysis Stress Test Kit (Agilent Technologies) using the Seahorse XF96 Analyzer.^34^ Cells were seeded in XF96-well plates at a density of 1.5 × 10^4^ cells per well and incubated overnight. After treatment, the culture medium was replaced with 180 μL per well of Seahorse XF Base Medium supplemented with 2 mM l-glutamine. The assay was performed by sequential injection of 10 mM glucose (a glycolysis substrate), 1 μM oligomycin (an ATP synthase inhibitor), and 50 mM 2-deoxy-d-glucose (a glycolysis inhibitor). Glycolytic activity and capacity were determined by analyzing alterations in the extracellular acidification rate (ECAR), including glycolysis and glycolytic capacity.

### 2.10 mRNA Levels by Quantitative Real-Time PCR (RT-qPCR)

The mRNA expression levels of genes associated with mitochondrial function, apoptosis, and ferroptosis were quantified using quantitative real-time PCR (RT-qPCR).^35^ Total RNA was extracted from H9c2 cells using the GeneJET RNA Purification Kit (Thermo Fisher Scientific). mRNA expression levels were quantified using the KAPA SYBR FAST One-Step RT-qPCR Kit (KAPA Biosystems, Boston, MA, USA) on a CFX96 Real-Time PCR Detection System (Bio-Rad, Hercules, CA, USA). Primers were synthesized by GenScript (Piscataway, NJ, USA). Gene expression data were normalized to glyceraldehyde-3-phosphate dehydrogenase (GAPDH), and relative expression levels were calculated using the 2^–ΔΔCt^ method. The primer sequences utilized in this study are provided in Table 1.

**Table 1:** List of Primer Sequences (Rat)

name		sequence (5′–3′)
ACSL4	sense	5′-ATTCCAGCACGAACGTCCAC-3′
	antisense	5′-CTGGGCATGGCTGCTGTTTT-3′
BAX	sense	5′-CTGCAGAGGATGATTGCTGA-3′
	antisense	5′-GATCAGCTCGGGCACTTTAG-3′
BCL2	sense	5′-GCTACGAGTGGGATACTGG-3′
	antisense	5′-GTGTGCAGATGCCGGTTCA-3′
DNM1	sense	5′-ATCCAGCTGCCTCAGATTGT-3′
	antisense	5′-GTGACCACACCAGTCCCTCT-3′
GPX4	sense	5′-TCATTCCCACCCTTTCCCTC-3′
	antisense	5′-GATACGCCGAGTGTGGTTTAC-3′
OPA1	sense	5′-TTGGGAGACCCTACAAGACG-3′
	antisense	5′-GTCTTCTGCGAAGTCGTTCC-3′
PGC1α	sense	5′-ATGTGTCGCCTTCTTGCTCT-3′
	antisense	5′-CGAGAAAAGGATCTCGAACG-3′
SIRT1	sense	5′-TCACCCTTCCTTCCTTCCTTCC-3′
	antisense	5′-GCTAGTTCAGTTGCCCTGCTGT-3′
SIRT3	sense	5′-AACACCTTTCCCTCCTCACACC-3′
	antisense	5′-GGGATCAAACTCGGCTTGTCAG-3′
GAPDH	sense	5′-GTGGACCTCATGGCCTACAT-3′
	antisense	5′-TGTGAGGGAGATGCTCAGTG-3′

### 2.11 Protein Expression by Western Blotting

Western blotting was utilized to evaluate the expression of proteins involved in mitochondrial integrity, apoptosis, and iron-dependent lipid peroxidation.^35^ Total protein was extracted from H9c2 cells using RIPA buffer supplemented with a protease inhibitor cocktail (Thermo Fisher Scientific). Protein concentration was quantified using the BCA Protein Assay Kit (Thermo Fisher Scientific). Equal amounts of protein (30 μg per sample) were separated by 10% SDS-PAGE and transferred onto PVDF membranes (Bio-Rad). Membranes were subsequently blocked and incubated overnight at 4 °C with primary antibodies targeting SIRT1 (Santa Cruz Biotechnology, Santa Cruz, CA, USA; sc-74465), SIRT3 (sc-365175), ACSL4 (sc-365230), GPX4 (sc-166570), DNM1 (sc-12724), OPA1 (sc-393296), PGC1α (sc-518025), BCL2 (Cell Signaling Technology, Danvers, MA, USA; cst#28150), BAX (cst#2772), and GAPDH (cst#5174). Membranes were then incubated with HRP-conjugated secondary antirabbit (sc-2357) and antimouse (sc-516102) antibodies. Protein bands were visualized using SignalFire ECL reagent (Cell Signaling Technology) and imaged on the iBright FL1500 Imaging System (Thermo Fisher Scientific). Band intensities were determined with ImageJ software, and results were normalized to GAPDH levels.

### 2.12 Statistical Analysis

All results are presented as the mean ± standard deviation (SD), calculated from at least four independent experiments. Statistical comparisons among groups were conducted using either one-way analysis of variance (ANOVA) followed by Tukey’s post hoc test or Student’s *t*-test, depending on which was most appropriate. Data analysis was performed using GraphPad Prism software (version 8.0.2; GraphPad Software, San Diego, CA, USA). Differences were considered statistically significant at a threshold of *p* < 0.05.

## 3 Results

### 3.1 Dapagliflozin Mitigates Doxorubicin-Induced Cellular Injuries and Metabolic Disturbances in H9c2 Cardiomyoblasts

The cardioprotective role of dapagliflozin was evaluated in a model of doxorubicin-induced acute cardiac damage. Exposure to doxorubicin (0.1 μM) significantly decreased cell viability, confirming its cytotoxic effects. Pretreatment with dapagliflozin prior to doxorubicin exposure restored cell viability to levels comparable to the control group (Figure 1A). Caspase-3/7 activity was markedly elevated by doxorubicin compared to the control, consistent with apoptotic activation. Dapagliflozin alone had no significant effect on caspase activity, but its preexposure substantially attenuated doxorubicin-triggered caspase activation (Figure 1B). Likewise, flow cytometric analysis of Annexin V/PI-stained cells demonstrated that doxorubicin led to a substantial increase in the population of early apoptotic (Q2) cells. In contrast, dapagliflozin caused a slight reduction in Q2 proportions compared to the control group under basal conditions, whereas initial exposure to dapagliflozin reduced the apoptotic population in cells treated with doxorubicin, suggesting its antiapoptotic potentials (Figure 1C).

**Figure 1:**
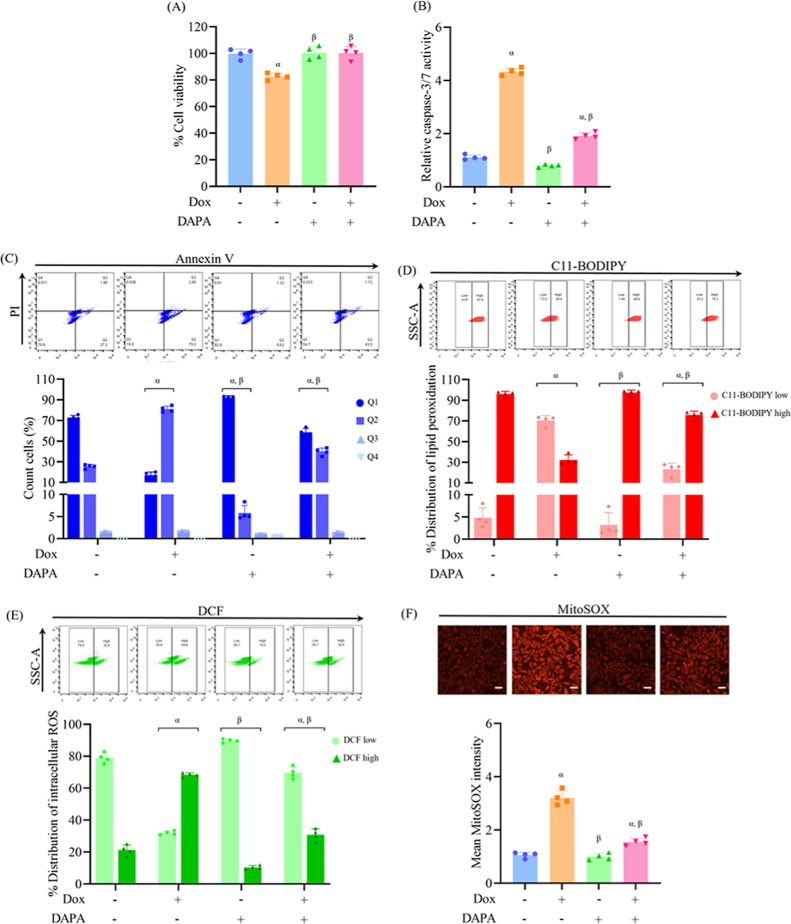
Dapagliflozin protects H9c2 cardiomyoblasts against doxorubicin-induced apoptosis, lipid peroxidation, and oxidative stress. H9c2 cells were treated with doxorubicin (Dox, 0.1 μM), dapagliflozin (DAPA, 10 μM), or pretreated with DAPA prior to Dox exposure. (A) Cell viability was assessed using the MTT assay. (B) Caspase-3/7 activity was measured to evaluate apoptotic response. (C) Flow cytometry using Annexin V-FITC/PI staining determined the proportions of viable, early apoptotic, late apoptotic, and necrotic cells. (D) Lipid peroxidation, a characteristic feature of ferroptosis, was measured using the C11-BODIPY probe. (E, F) Intracellular and mitochondrial ROS production was quantified using DCFH-DA and MitoSOX Red staining, respectively. Results are expressed as mean ± SD (*n* = 4). ^α^ *p* < 0.05 vs control, ^β^ *p* < 0.05 vs Dox. Scale bar: 50 μm.

To evaluate the involvement of ferroptosis, lipid peroxidation was measured using the C11-BODIPY probe. Treatment with doxorubicin raised lipid peroxidation levels, reflecting the induction of ferroptosis. Dapagliflozin, as a single agent, had no effect on lipid peroxidation; however, its preincubation suppressed doxorubicin-induced lipid peroxidation, highlighting its inhibitory response on ferroptosis (Figure 1D). Oxidative stress was further assessed by measuring intracellular ROS and mitochondrial ROS levels. Doxorubicin exposure elevated both cytosolic and mitochondrial ROS generation. Dapagliflozin did not alter ROS levels under basal conditions; however, pretreatment considerably attenuated ROS accumulation in doxorubicin-exposed cells, emphasizing its antioxidant potential under stress conditions (Figure 1E,F).

Mitochondrial function was evaluated and found to be markedly impaired by doxorubicin, affecting mitochondrial respiratory parameters, including basal respiration, ATP production, maximal respiration, and spare respiratory capacity. In the absence of doxorubicin stress, dapagliflozin enhanced mitochondrial respiratory function, suggesting a potential bioenergetic benefit. Importantly, preincubation with dapagliflozin restored mitochondrial function under pathological conditions, demonstrating a protective effect against doxorubicin-induced mitochondrial dysfunction (Figure 2A). Similarly, doxorubicin suppressed both glycolysis and glycolytic capacity. Dapagliflozin administered alone had no impact on glycolysis under nonstressed conditions, but its pretreatment restored glycolytic function in doxorubicin-exposed cells, underscoring its ability to preserve metabolic flexibility under chemotherapeutic stress (Figure 2B).

**Figure 2:**
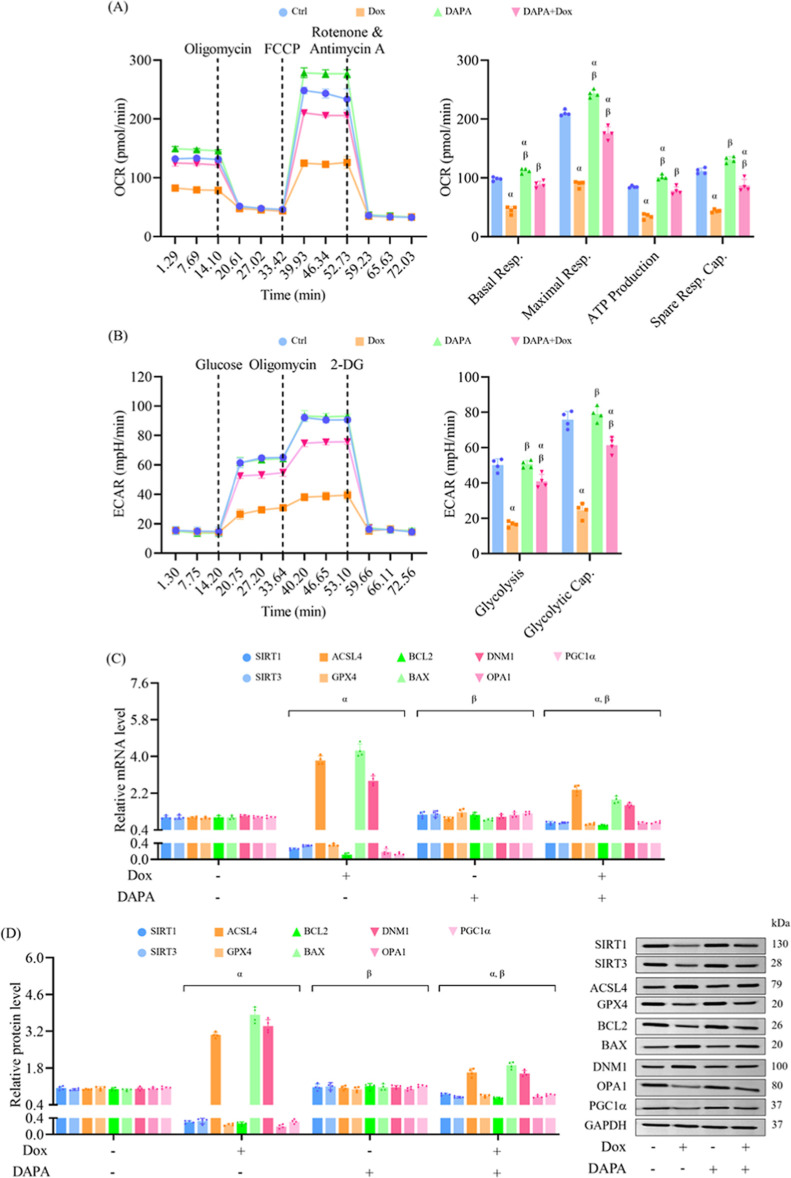
Dapagliflozin improves mitochondrial function and modulates molecular markers of cellular metabolism and injury in doxorubicin-treated cells. H9c2 cells were treated as described in Figure 1. (A) Mitochondrial respiration parameters, including basal respiration, maximal respiration, ATP production, and spare respiratory capacity, were evaluated using the Seahorse XF Mito Stress Test. (B) Glycolytic function, including glycolysis and glycolytic capacity, was assessed using the Seahorse XF Glycolysis Stress Test. (C) Relative mRNA expression of genes involved in sirtuin signaling (SIRT1, SIRT3), ferroptosis (ACSL4, GPX4), apoptosis (BCL2, BAX), and mitochondrial dynamics/metabolism (DNM1, OPA1, PGC1α) were quantified using RT-qPCR. (D) Western blot analysis was used to evaluate protein expression of key regulators involved in these pathways. Results are expressed as mean ± SD (*n* = 4). ^α^ *p* < 0.05 vs control, ^β^ *p* < 0.05 vs Dox.

To explore underlying mechanisms, gene and protein expression profiles associated with mitochondrial function, apoptosis, and ferroptosis were examined. Exposure to doxorubicin downregulated SIRT1, SIRT3, GPX4, BCL2, OPA1, and PGC1α, while upregulating ACSL4, BAX, and DNM1. Prechallenge with dapagliflozin reversed these changes, indicating the activation of prosurvival and mitochondrial regulatory pathways, as well as the suppression of proapoptotic and ferroptotic signaling (Figure 2C). These transcriptional changes were corroborated by Western blot analysis, which reflected consistent protein expression trends (Figure 2D). Taken together, these findings indicate that dapagliflozin mitigates cardiotoxicity due to doxorubicin by inhibiting apoptosis, oxidative stress, and ferroptosis, while preserving mitochondrial respiration and glycolytic capacity. The observed regulation of SIRT1/SIRT3 and ferroptosis-related markers such as GPX4 and ACSL4 suggests that these pathways play a central role in mediating the cardioprotective properties of dapagliflozin.

### 3.2 SIRT1 and SIRT3 Inhibition Attenuates the Cardioprotective Effects of Dapagliflozin in Doxorubicin-Induced H9c2 Toxicity

To further determine the involvement of SIRT1 and SIRT3 in mediating the protective potentials of dapagliflozin under doxorubicin-triggered injury, cells were pretreated with selective SIRT signaling inhibitors, Ex-527 (SIRT1 inhibitor, 1 μM) or 3-TYP (SIRT3 inhibitor, 5 μM), followed by treatment with dapagliflozin and doxorubicin. Cell viability analysis demonstrated that the restoration of viability by dapagliflozin in doxorubicin-treated cells was compromised by either SIRT1 or SIRT3 blockade, suggesting that the full protective effect of dapagliflozin against doxorubicin-induced cytotoxicity requires both SIRT1 and SIRT3 activity (Figure 3A). In addition, the reduction in caspase-3/7 activity observed with dapagliflozin and doxorubicin combined treatment was reversed in the presence of either inhibitor (Figure 3B). Flow cytometry supported this, showing increased early apoptotic (Q2) populations in EX-527 and 3-TYP pretreated groups compared to the dapagliflozin and doxorubicin group (Figure 3C), confirming that SIRT1 and SIRT3 activities are necessary for the antiapoptotic response of dapagliflozin. Lipid peroxidation analysis revealed that SIRT1 or SIRT3 inhibition reversed the suppressive effect of dapagliflozin, leading to elevated lipid peroxidation in doxorubicin-exposed cells (Figure 3D). Furthermore, the antioxidant impact of dapagliflozin in doxorubicin-exposed cells was attenuated in the presence of EX-527 or 3-TYP, as indicated by elevated intracellular and mitochondrial ROS levels (Figure 3E,F).

**Figure 3:**
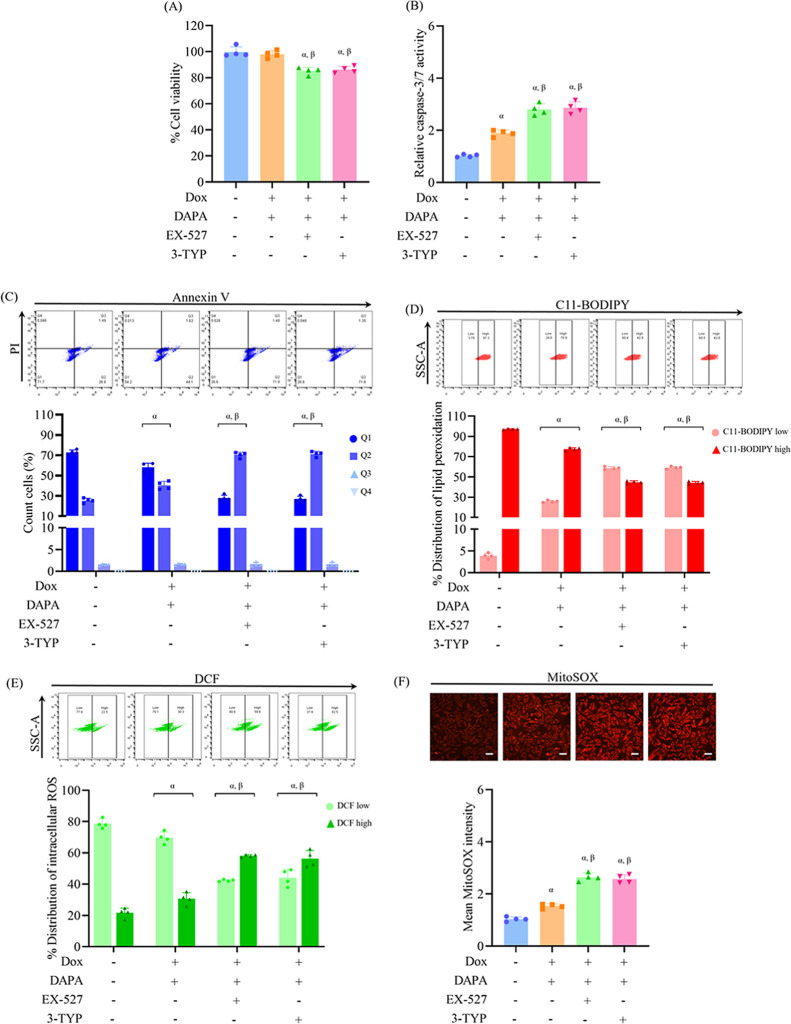
Pharmacological inhibition of SIRT1 or SIRT3 diminishes the protective effects of dapagliflozin against doxorubicin-induced cardiotoxicity. H9c2 cells were treated with dapagliflozin (DAPA, 10 μM) before doxorubicin (Dox, 0.1 μM) exposure, or pretreated with EX-527 (SIRT1 inhibitor, 1 μM) or 3-TYP (SIRT3 inhibitor, 5 μM), followed by DAPA before Dox exposure. (A) Cell viability was evaluated via MTT assay. (B) Apoptosis was assessed by measuring caspase-3/7 activity. (C) Flow cytometric analysis with Annexin V-FITC/PI staining distinguished stages of cell death. (D) Lipid peroxidation was quantitatively assessed using the C11-BODIPY fluorescent probe. (E, F) Levels of intracellular and mitochondrial ROS were detected using DCFH-DA and MitoSOX Red, respectively. Results are expressed as mean ± SD (*n* = 4). ^α^ *p* < 0.05 vs control, ^β^ *p* < 0.05 vs DAPA + Dox, ^γ^ *p* < 0.05 vs EX-527 + DAPA + Dox. Scale bar: 50 μm.

Seahorse analysis showed that the restoration of basal respiration, ATP production, maximal respiration, and spare respiratory capacity by dapagliflozin in cells exposed to doxorubicin was markedly impaired upon SIRT1 or SIRT3 inhibition (Figure 4A). Similarly, the glycolysis and glycolytic capacity restored by dapagliflozin under pathological conditions were also reduced in the presence of either EX-527 or 3-TYP, suggesting a broader metabolic role for SIRT1 and SIRT3 in mediating the protective effects of dapagliflozin (Figure 4B). Gene expression profiles indicated that the dapagliflozin-mediated regulation of target genes in doxorubicin-treated cells, including SIRT1, SIRT3, GPX4, ACSL4, BCL2, BAX, DNM1, OPA1, and PGC1α, was attenuated upon treatment with either inhibitor (Figure 4C). Western blot analysis confirmed these trends at the protein levels, showing altered expression profiles consistent with reduced dapagliflozin efficacy in the presence of SIRT1 or SIRT3 blockade (Figure 4D). Collectively, these findings demonstrate that SIRT1 and SIRT3 are essential mediators underlying the cardioprotective potential of dapagliflozin in doxorubicin-mediated cellular injuries. The lack of significant differences between the two inhibitors suggests potential functional overlap or compensatory crosstalk between SIRT1 and SIRT3 in regulating cellular stress responses during chemotherapeutic challenges.

**Figure 4:**
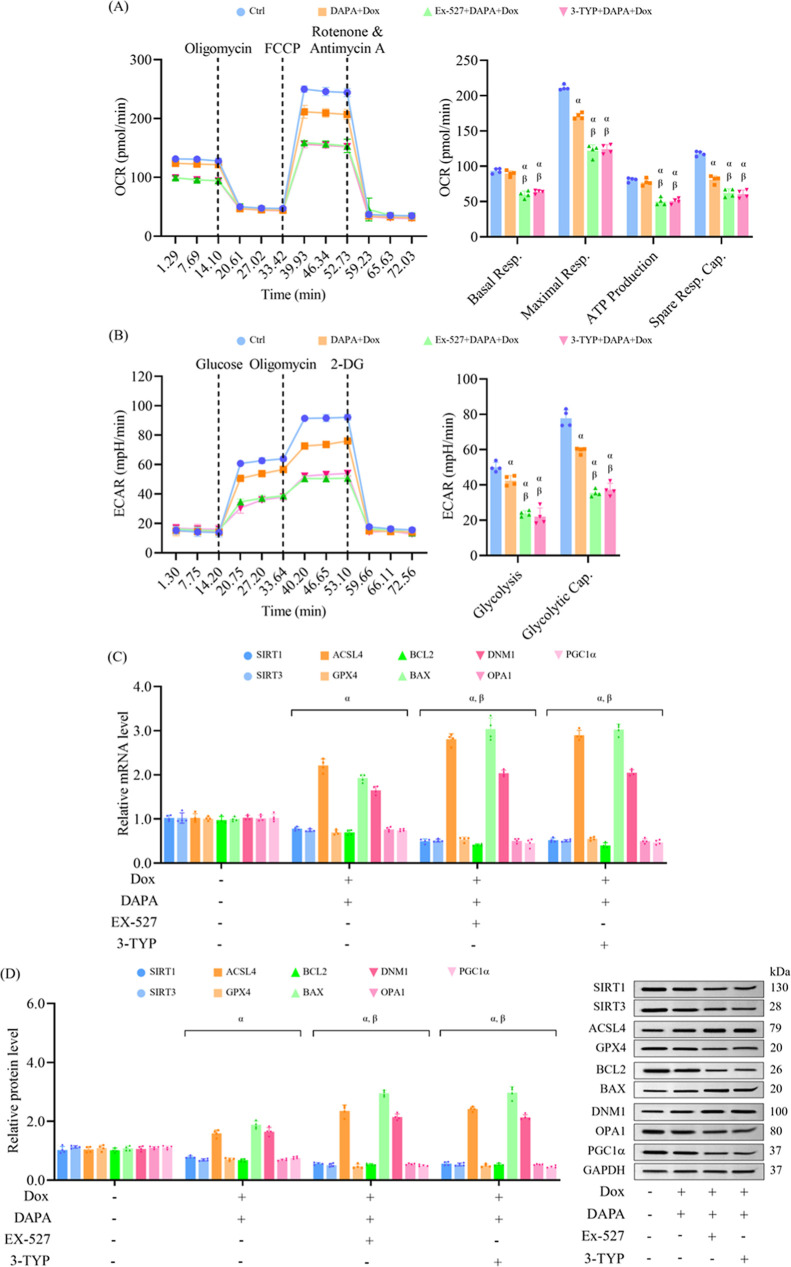
SIRT1 or SIRT3 inhibition impairs the ability of dapagliflozin to enhance mitochondrial bioenergetics and mitigate cellular injuries in doxorubicin-challenged cells. H9c2 cells were treated as mentioned in Figure 3. (A) Mitochondrial respiration metrics were assessed via Seahorse XF Mito Stress Test. (B) Glycolytic function was evaluated with the Seahorse XF Glycolysis Stress Test. (C) Transcript levels of key genes related to sirtuin signaling, ferroptosis, apoptosis, and mitochondrial function were determined by RT-qPCR. (D) Protein markers associated with these pathways were assessed by Western blotting. Results are expressed as mean ± SD (*n* = 4). ^α^ *p* < 0.05 vs control, ^β^ *p* < 0.05 vs DAPA + Dox, ^γ^ *p* < 0.05 vs EX-527 + DAPA + Dox.

### 3.3 Modulation of Ferroptosis Alters the Cardioprotective Impacts of Dapagliflozin under Doxorubicin-Induced Stress

We next examined the contribution of ferroptosis to the cardiac defensive mechanism of dapagliflozin by employing the ferroptosis inhibitor ferrostatin-1 (Fer-1, 1 μM) or the ferroptosis inducer erastin (3 μM). Cell viability analysis showed that pretreatment with Fer-1 enhanced the protective impact of dapagliflozin in doxorubicin-exposed cells, while exposure to erastin reduced cell viability compared to cells pretreated with dapagliflozin prior to doxorubicin (Figure 5A). These results suggest that ferroptosis inhibition potentiates, and ferroptosis activation compromises, the protective efficacy of dapagliflozin under chemotherapeutic stress in cardiac cells. Moreover, the reduction in caspase-3/7 activity observed in cells incubated with dapagliflozin and doxorubicin was further reduced by Fer-1 pretreatment, whereas erastin pretreatment increased caspase activity, thereby counteracting the antiapoptotic effect of dapagliflozin (Figure 5B). A comparable trend was observed by flow cytometry, with Fer-1 pretreatment reducing the early apoptotic (Q2) population in cells treated with dapagliflozin and doxorubicin, while prior incubation with erastin elevated Q2 relative to the dapagliflozin and doxorubicin group (Figure 5C), indicating that ferroptosis influences apoptotic susceptibility under dapagliflozin-mediated protection from doxorubicin-triggered injury. C11-BODIPY analysis revealed that Fer-1 preexposure further suppressed lipid peroxidation in cells exposed to dapagliflozin and doxorubicin, whereas initial treatment with erastin reversed this effect, resulting in a significant elevation of lipid peroxidation levels (Figure 5D). Consistently, both intracellular and mitochondrial ROS, which were lowered by dapagliflozin in doxorubicin-challenged cells, were further diminished by Fer-1 and increased by erastin (Figure 5E,F).

**Figure 5:**
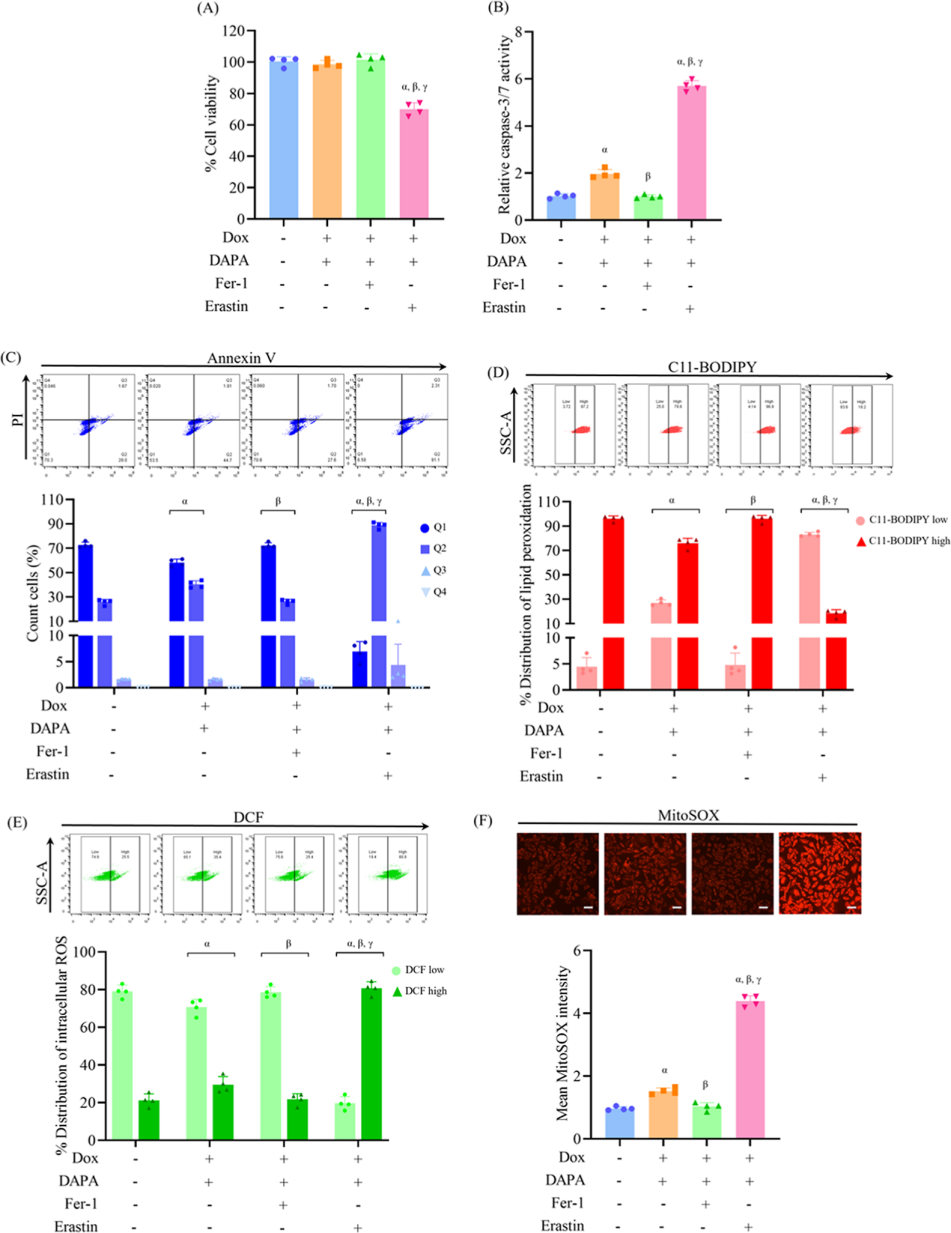
Ferroptosis modulates dapagliflozin’s cardioprotective potentials in doxorubicin-treated cells. H9c2 cells were treated with dapagliflozin (DAPA, 10 μM) before doxorubicin (Dox, 0.1 μM) exposure, or pretreated with either Fer-1 (ferroptosis inhibitor, 1 μM) or erastin (ferroptosis inducer, 3 μM), followed by DAPA and then Dox. (A) Cell viability was assessed by MTT assay. (B) Caspase-3/7 activity was analyzed to measure apoptosis. (C) Apoptotic cell populations were evaluated using Annexin V-FITC/PI flow cytometry. (D) Lipid peroxidation was quantified with C11-BODIPY staining. (E, F) ROS levels were quantified using the fluorescent probes: DCFH-DA for the overall intracellular pool and MitoSOX Red specifically for the mitochondrial component. Results are expressed as mean ± SD (*n* = 4). ^α^ *p* < 0.05 vs control, ^β^ *p* < 0.05 vs DAPA + Dox, ^γ^ *p* < 0.05 vs Fer-1 + DAPA + Dox. Scale bar: 50 μm.

Cellular bioenergetic analysis showed that Fer-1 treatment before dapagliflozin and doxorubicin improved maximal respiration and spare respiratory capacity. In contrast, erastin pretreatment impaired all major respiratory parameters, including basal respiration, ATP production, and maximal respiration, indicating a detrimental impact of ferroptosis activation on mitochondrial bioenergetics (Figure 6A). Glycolytic function followed a similar pattern, with ferroptosis inhibition enhancing glycolysis and glycolytic capacity, while ferroptosis activation impaired them (Figure 6B), indicating that ferroptosis disrupts dapagliflozin-mediated energy metabolism under doxorubicin-triggered stress. mRNA and protein expression profiling demonstrated that dapagliflozin-mediated regulation of SIRT1, SIRT3, GPX4, ACSL4, BCL2, BAX, DNM1, OPA1, and PGC1α in doxorubicin-treated cells was further amplified by Fer-1 and diminished by erastin. Notably, SIRT1 and SIRT3 expression were also modulated when Fer-1 or erastin was applied before dapagliflozin in doxorubicin-treated cells, implying that ferroptotic signaling may feedback to influence sirtuin expression under chemotherapeutic challenge (Figure 6C,D). Overall, these findings indicate that inhibition of ferroptosis amplifies, whereas activation of ferroptosis diminishes, the cardioprotective effects of dapagliflozin in cells exposed to doxorubicin. Thus, ferroptosis serves as a key mediator of doxorubicin-induced cardiotoxicity and represents a critical target through which dapagliflozin exerts its protective mechanism under chemotherapeutic stress.

**Figure 6:**
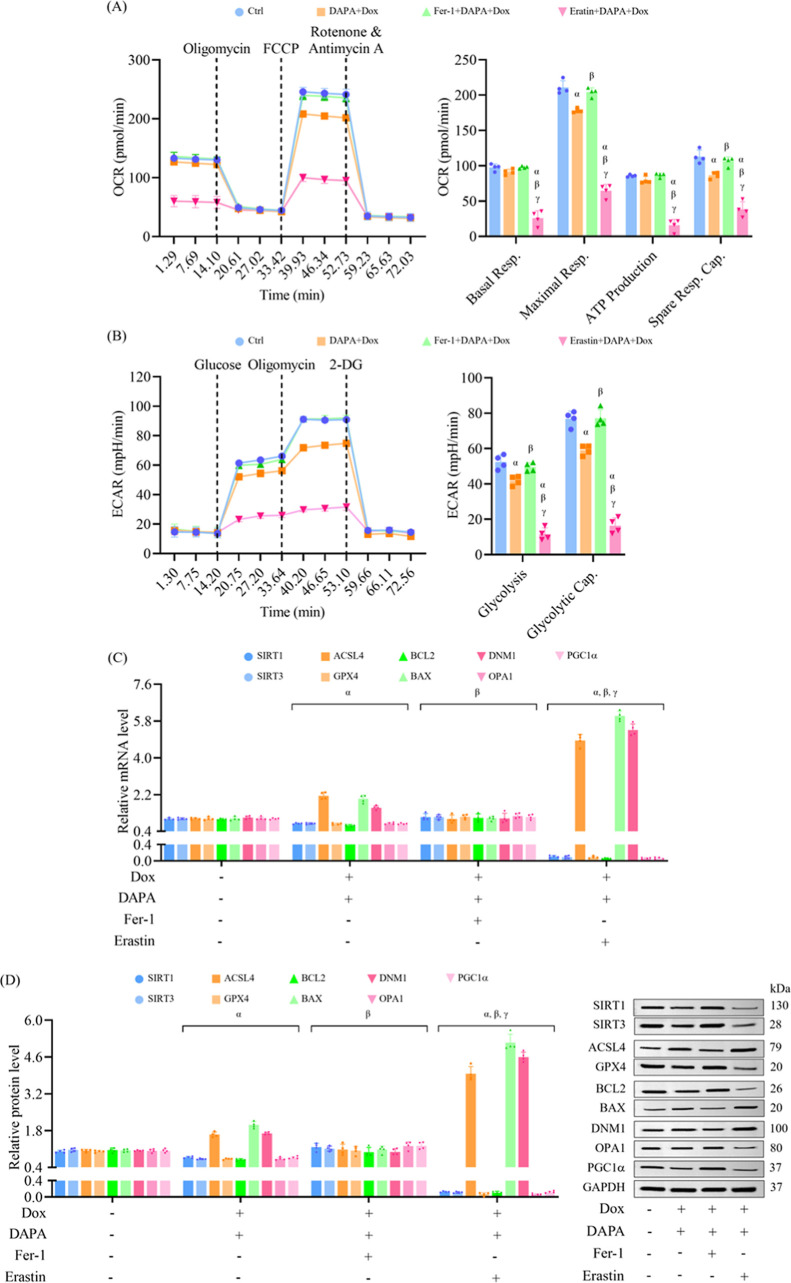
Ferroptosis inhibition enhances dapagliflozin-induced metabolic and molecular adaptations in doxorubicin-exposed cells. H9c2 cells were treated as indicated in Figure 5. (A) Mitochondrial respiratory profile was determined by Seahorse XF Mito Stress Test. (B) Glycolytic capacity was evaluated using Seahorse XF Glycolysis Stress Test. (C) mRNA expression of markers related to sirtuin activity, ferroptosis regulation, apoptosis, and mitochondrial dynamics was assessed by RT-qPCR. (D) Western blotting detected and quantified key proteins associated with these pathways. Results are expressed as mean ± SD (*n* = 4). ^α^ *p* < 0.05 vs control, ^β^ *p* < 0.05 vs DAPA + Dox, ^γ^ *p* < 0.05 vs Fer-1 + DAPA + Dox.

### 3.4 Suppression of SIRT1 or SIRT3 Compromises Dapagliflozin-Mediated Cardiac Defense in Doxorubicin-Treated H9c2 Cells, Irrespective of Ferroptosis Blockade

To further clarify the mechanistic interplay between sirtuin signaling and ferroptosis in dapagliflozin-mediated cardiac defense under doxorubicin injury, cells underwent sequential pretreatment with EX-527 or 3-TYP and Fer-1, followed by dapagliflozin treatment prior to doxorubicin. For cell viability, Fer-1 enhanced dapagliflozin-mediated protection against doxorubicin cytotoxicity; however, this effect was diminished when either SIRT1 or SIRT3 was inhibited before Fer-1 coincubation (Figure 7A). Caspase-3/7 activity assay showed that the apoptotic suppression conferred by Fer-1 in cells treated with combined dapagliflozin and doxorubicin was largely abolished by either EX-527 or 3-TYP, resulting in increased apoptotic signaling comparable to cells without ferroptosis inhibition (Figure 7B). Flow cytometric analysis of apoptotic cell populations corroborated these findings, with higher proportions of early apoptotic (Q2) cells detected when SIRT1 or SIRT3 was blocked, despite the presence of Fer-1 in dapagliflozin plus doxorubicin-treated cells (Figure 7C). Similarly, the suppression of lipid peroxidation achieved by Fer-1 in cells preexposed to dapagliflozin before doxorubicin challenge was reversed when either EX-527 or 3-TYP was applied, indicating that blockade of SIRT1 or SIRT3 reinstates ferroptotic stress even under conditions of pharmacological ferroptosis suppression (Figure 7D). Assessment of intracellular and mitochondrial ROS revealed that Fer-1 potentiated the antioxidant effects of dapagliflozin in doxorubicin-damaged cardiomyoblasts, but these benefits were markedly suppressed by either SIRT1 or SIRT3 blockade (Figure 7E,F).

**Figure 7:**
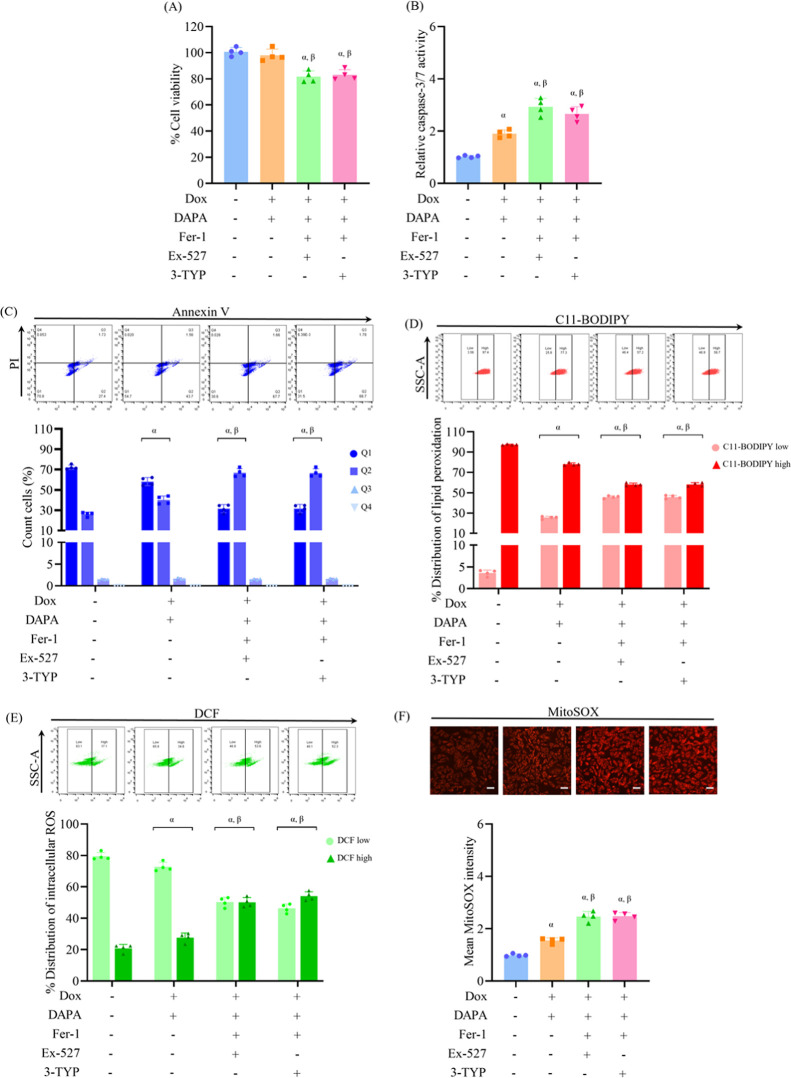
SIRT1 and SIRT3 are required for ferroptosis inhibition-mediated cardioprotective effects of dapagliflozin in doxorubicin-treated cells. H9c2 cells were treated with dapagliflozin (DAPA, 10 μM) before doxorubicin (Dox, 0.1 μM), or sequentially pretreated with EX-527 or 3-TYP, followed by Fer-1, then DAPA, and finally exposed to Dox. (A) Cell viability was determined by MTT assay. (B) Caspase-3/7 activity was measured to assess apoptosis. (C) Apoptotic populations were identified by flow cytometry with Annexin V-FITC/PI staining. (D) Lipid peroxidation was detected using C11-BODIPY fluorescence. (E, F) Intracellular and mitochondrial ROS levels were measured by DCFH-DA and MitoSOX Red staining, respectively. Results are expressed as mean ± SD (*n* = 4). ^α^ *p* < 0.05 vs control, ^β^ *p* < 0.05 vs DAPA + Dox, ^γ^ *p* < 0.05 vs EX-527 + Fer-1 + DAPA + Dox. Scale bar: 50 μm.

Assessment of mitochondrial bioenergetics revealed that the improvements in basal respiration, ATP production, maximal respiration, and spare respiratory capacity achieved by combined dapagliflozin and Fer-1 treatment in doxorubicin-challenged cells were severely impaired by SIRT1 or SIRT3 inhibition (Figure 8A). Likewise, glycolytic function, which was preserved and even enhanced by Fer-1 in cells treated with both dapagliflozin and doxorubicin, was reduced upon inhibition of either sirtuin (Figure 8B). At the transcriptional level, blockade of SIRT1 or SIRT3 reversed the protective responses of dapagliflozin and Fer-1 in doxorubicin-challenged cells, as reflected by altered expression of genes involved in sirtuin signaling (SIRT1, SIRT3), ferroptosis (GPX4, ACSL4), apoptosis (BCL2, BAX), and mitochondrial dynamics (DNM1, OPA1, PGC1α) (Figure 8C). Parallel changes in protein levels were noted, confirming that the protective molecular effects of dapagliflozin were diminished by inhibition of either sirtuin, notwithstanding ferroptosis inhibition (Figure 8D). Collectively, these data establish that SIRT1 and SIRT3 function as critical upstream regulators necessary for dapagliflozin to exert its full cardioprotective potentials against doxorubicin-induced injury. Notably, inhibition of either sirtuin compromises the ability of dapagliflozin to suppress ferroptosis and associated oxidative and apoptotic stress, even in the presence of the potent ferroptosis inhibitor, indicating that sirtuin signaling is indispensable for integrating ferroptosis regulation into dapagliflozin’s cardiac defensive mechanisms.

**Figure 8:**
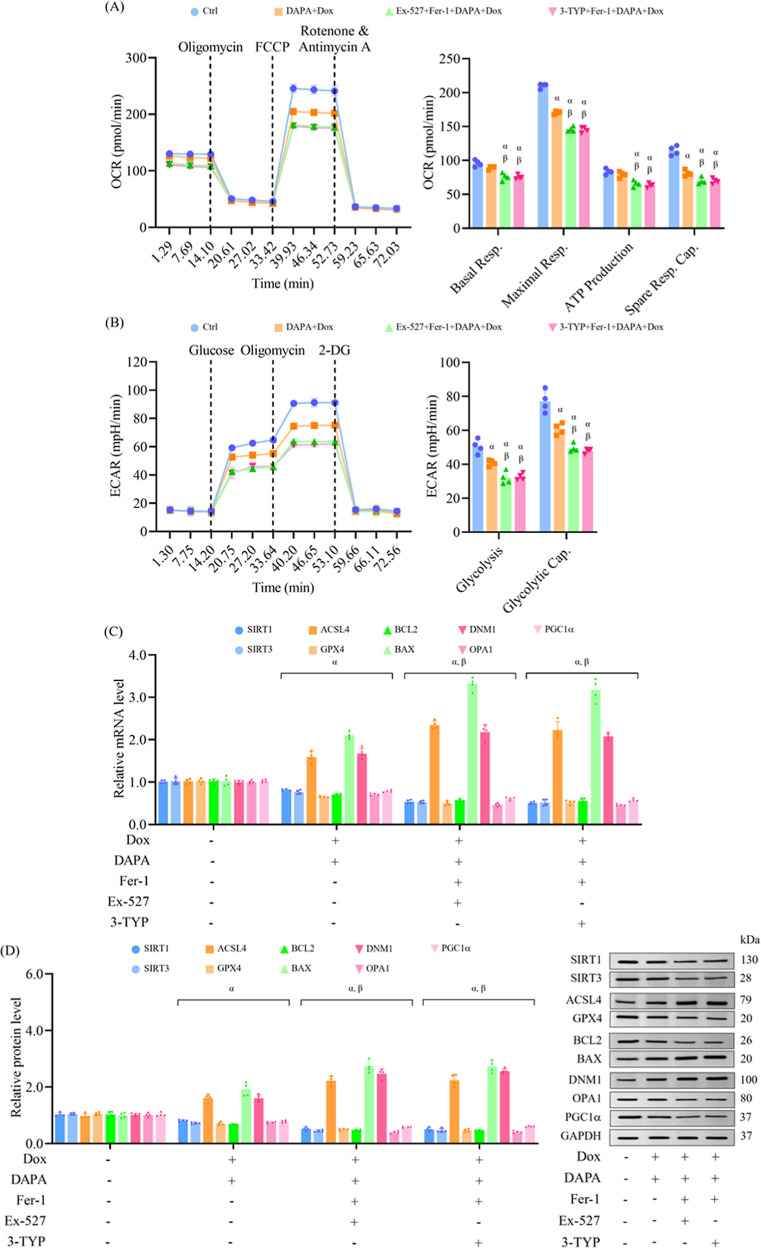
SIRT1 and SIRT3 are essential for dapagliflozin to enhance mitochondrial function and prevent ferroptosis-mediated cellular injury in doxorubicin-treated cells. H9c2 cells were treated as described in Figure 7. (A) Cellular bioenergetics in live cells were recorded using the Seahorse XF Mito Stress Test. (B) Glycolytic profile was measured using the Seahorse XF Glycolysis Stress Test. (C) Gene expression profiling of sirtuin, ferroptosis, apoptotic, and mitochondrial dynamics-related markers was conducted using RT-qPCR. (D) Western blotting examined protein expression changes of key regulators involved in these pathways. Results are expressed as mean ± SD (*n* = 4). ^α^ *p* < 0.05 vs control, ^β^ *p* < 0.05 vs DAPA + Dox, ^γ^ *p* < 0.05 vs EX-527 + Fer-1 + DAPA + Dox.

## 4 Discussion

Heart failure remains a major cause of morbidity and mortality and is often exacerbated by chemotherapeutic agents such as doxorubicin. Doxorubicin-induced cardiotoxicity restricts its clinical application and is characterized by progressive mitochondrial dysfunction, oxidative stress, and activation of regulated cell death pathways, which together lead to cardiac dysfunction and promote the progression of heart failure.^19–21^ Recent mechanistic studies have identified mitochondrial dysfunction as a central pathological hub linking chemotherapeutic stress to myocardial injury, underscoring mitochondria as key therapeutic targets in cardiotoxicity.^36,37^ The present work provides the first evidence that dapagliflozin protects cardiomyocytes from doxorubicin-triggered cellular damage through activation of SIRT1/SIRT3 signaling and inhibition of ferroptosis. This dual regulation provides a novel mechanistic link between mitochondrial regulation and cell death control, highlighting the SIRT1/SIRT3-ferroptosis axis as a potential therapeutic target for cardioprotection during chemotherapy.

Sirtuins are NAD^+^-dependent deacetylases that function as key regulators of mitochondrial biogenesis and oxidative metabolism. Among these, SIRT1 and SIRT3 have essential roles in cardiac protection through activation of antioxidant and survival pathways.^38,39^ In this study, doxorubicin exposure downregulated SIRT1 and SIRT3, resulting in decreased PGC-1α, OPA1, and BCL2 levels alongside elevated BAX and DNM1 expression, collectively indicating impaired mitochondrial biogenesis and activation of apoptotic signaling. Dapagliflozin reversed these effects, suggesting that SIRT1 and SIRT3 activation contribute to mitochondrial stabilization and antiapoptotic regulation. Previous findings demonstrated that SIRT1 activation alleviates oxidative stress by modulating stress-related kinase pathways, particularly those involving the MAPK family and protein kinase B (Akt).^40^ In addition, SIRT3 preserves mitochondrial structure and energy metabolism during doxorubicin exposure by suppressing the stress-related protein BNIP3 and maintaining the mitochondrial complex of cytochrome c oxidase I and uncoupling protein 3 (UCP3), which is crucial for oxidative stress defense.^23^ Emerging evidence further indicates that other sirtuins, particularly SIRT5, contribute to mitochondrial stress adaptation by coordinating mitochondria-endoplasmic reticulum unfolded protein response and mitophagy signaling in models of cardiovascular injury.^41,42^ Therefore, the restoration of both nuclear and mitochondrial sirtuins by dapagliflozin is central to the recovery of cellular metabolism and resistance to oxidative stress.

Pharmacological inhibition of sirtuins in the present study further clarified their essential role in the cardioprotective mechanism of dapagliflozin. When either SIRT1 or SIRT3 was inhibited, dapagliflozin failed to restore cell viability, mitochondrial function, or redox stability in cardiomyocytes injured by doxorubicin, indicating that both sirtuins are indispensable upstream mediators that coordinate cellular adaptation under chemotherapeutic stress. Prior studies have shown that loss of SIRT3 leads to the accumulation of acetylated mitochondrial proteins, including electron transport chain complexes I, II, and V as well as SOD2, and development of dilated cardiomyopathy during doxorubicin exposure.^43,44^ Suppression of SIRT1 increased oxidative stress and apoptosis in cardiac tissue by inhibiting the antioxidant response pathway, as evidenced by decreased expression of SOD1 and BCL2, increased expression of BAX, and enhanced phosphorylation of p38 MAPK, thereby promoting caspase-3-mediated apoptosis.^22^ Together, the activation of both SIRT1 and SIRT3 forms the core protective mechanism potentiated by dapagliflozin.

Ferroptosis, a form of regulated cell death driven by lipid peroxidation and iron accumulation, has emerged as a crucial mechanism in doxorubicin-induced cardiac injury. The accumulation of lipid peroxides damages cell membranes and triggers metabolic collapse.^45–47^ The present study revealed that doxorubicin markedly increases lipid peroxidation and ACSL4 expression and reduces GPX4 levels, indicating activation of ferroptotic signaling. Earlier investigations demonstrated that ferroptosis contributes to cardiomyocyte death due to doxorubicin exposure through mitochondrial lipid peroxidation.^21^ Early exposure to dapagliflozin suppressed lipid peroxidation and restored GPX4 expression, suggesting inhibition of ferroptosis. In line with our findings, previous studies have shown that dapagliflozin prevents ferroptosis-related injury in cardiac cells by preserving antioxidant enzyme activity through upregulation of GPX4.^48,49^ Direct mitochondrial targeting suppresses regulated cell death, while lipid–selenium conjugates induce mitophagy and inhibit necroptosis via metabolic exhaustion, highlighting mitochondria as upstream regulators of ferroptosis-related injury.^50^ Collectively, suppression of ferroptosis constitutes a major component of the cardiac defensive mechanism of dapagliflozin. Furthermore, the contribution of ferroptosis in mediating the cardioprotective potentials of dapagliflozin was further confirmed through pharmacological modulation. Inhibition of ferroptosis with Fer-1 enhanced the protective effects of dapagliflozin, whereas activation of ferroptosis by erastin diminished them. In accordance with these findings, several studies have reported that inhibition of ferroptosis prevents mitochondrial oxidative stress and improves cardiac cell survival during doxorubicin treatment.^51,52^ Thus, suppression of ferroptosis by dapagliflozin can be considered a critical step in reducing oxidative damage and maintaining cell viability under chemotherapeutic stress.

A key discovery of this study is that activation of SIRT1 and SIRT3 signaling is essential for suppressing ferroptosis. Blocking either sirtuin eliminated the ability of dapagliflozin to prevent lipid peroxidation and loss of GPX4, even when ferroptosis was pharmacologically inhibited, indicating that sirtuin signaling acts upstream of ferroptosis and provides the metabolic environment necessary for antioxidant defense. Previous research has demonstrated that SIRT1 activation prevents ferroptosis by promoting antioxidant gene transcription via nuclear factor erythroid 2-related factor 2 (NRF2) and its sensor Kelch-like ECH-associated protein 1 (Keap1), as demonstrated in H9c2 cardiomyocytes and a cardiac-specific SIRT1 knockout mouse model, where SIRT1 enhances NRF2 nuclear translocation and activates downstream targets such as GPX4.^53–55^ A separate finding indicated that SIRT3 maintains GPX4 stability and lipid homeostasis under oxidative conditions.^56^ This current study expands upon previous findings, demonstrating that the simultaneous activation of both SIRT1 and SIRT3 is essential for the complete prevention of ferroptosis in cardiomyocytes during doxorubicin exposure.

The interaction between ferroptosis and sirtuin expression observed in the present study leads to the hypothesis that a regulatory feedback loop exists between these processes. Activation of ferroptosis by erastin reduced SIRT1 and SIRT3 expression, while inhibition of ferroptosis by Fer-1 increased their levels. This bidirectional relationship implies that oxidative lipid damage can suppress sirtuin signaling, while ferroptosis inhibition can stabilize sirtuin expression and function. Limiting lipid peroxidation was reported to upregulate sirtuin activity in cells by improving mitochondrial antioxidant capacity, as shown in cardiomyocyte-specific SIRT3 knockout mice, where loss of SIRT3 reduced mitochondrial antioxidant markers such as GPX4, which were restored by Fer-1 treatment.^57^ This reciprocal relationship between sirtuins and ferroptosis may represent an adaptive mechanism for controlling redox balance in cardiomyocytes exposed to metabolic and oxidative stress.

Mitochondrial impairment is recognized as a key contributor to doxorubicin-induced cardiac injury, as it disrupts energy production and promotes oxidative stress. Doxorubicin interferes with the electron transport chain, leading to excessive ROS production and ATP depletion.^58,59^ In the present study, exposure to doxorubicin markedly reduced mitochondrial respiration, ATP generation, and glycolytic capacity, confirming severe metabolic impairment. Research to date suggests that doxorubicin damages mitochondrial DNA and respiratory complexes, resulting in structural disorganization and loss of energy supply.^59^ Initial treatment with dapagliflozin restored mitochondrial respiration and energy production, indicating recovery of mitochondrial efficiency under stress conditions. In agreement with our data, earlier findings demonstrated that dapagliflozin reduced oxidative injury and preserved mitochondrial membrane potential in cardiac cells.^60,61^ In H9c2 cardiomyocytes, dapagliflozin mitigated oxidative stress, modulated apoptotic regulators (BCL-2, BAX), and preserved PGC-1α expression.^60^ In rodent hearts, it promoted PGC-1α, strengthened oxidative phosphorylation, and regulated mitochondrial dynamics (DRP1, MFN2, OPA1), collectively supporting mitochondrial biogenesis and functional integrity.^61^ Taken together, improvement of mitochondrial energy metabolism is a major mechanism underlying the cardioprotective potential of dapagliflozin.

Mitochondrial quality control and dynamics are also critical for maintaining cellular energy production and preventing apoptosis. Mitochondrial fusion protein OPA1 maintains cristae organization and supports efficient respiration in cardiomyocytes, whereas fission protein DNM1 promotes mitochondrial fragmentation under stress.^62–64^ Recent evidence links cytoskeletal regulation to mitochondrial dynamics in cardiac pathology, with microtubules governing the fission–fusion balance, while pathological activation of nuclear receptor subfamily 4 group A member 1 (NR4A1) aggravates ischemia/reperfusion injury by promoting mitochondrial fragmentation and inhibiting mitophagy.^65,66^ The current results show that early exposure to dapagliflozin upregulated OPA1 and downregulated DNM1, reflecting strengthened mitochondrial integrity and balanced dynamics. Previous studies reported that activation of SIRT3 enhances OPA1 activity and supports mitochondrial fusion during oxidative stress.^12,67^ Additional reports demonstrated that activation of PGC-1α increases antioxidant enzyme expression and reduces oxidative overload in the myocardium.^68,69^ These results support the view that dapagliflozin stabilizes mitochondrial morphology and biogenesis through activation of sirtuin-dependent pathways.

Apoptosis is an additional key process contributing to cardiotoxicity under doxorubicin exposure.^70^ The present findings show that cotreatment with dapagliflozin reduced caspase-3/7 activation and restored the balance between BCL2 and BAX expression, indicating inhibition of mitochondrial-mediated apoptosis. Similar pathways suppress septic myocardial apoptosis, as astragaloside IV activates dual specificity phosphatase 1 (DUSP1)-prohibitin-2 signaling to preserve mitochondrial integrity.^71^ Evidence from an earlier study reported that activation of SIRT1 prevented p53-dependent apoptotic signaling and protected cardiomyocytes against doxorubicin-induced injury.^9^ According to previous reports, SIRT3 activation protects against mitochondrial apoptosis by preserving antioxidant enzyme activity and reducing oxidative stress, including upregulating BCL-2, downregulating BAX, and lowering ROS production in H9c2 rat cardiomyoblasts,^72^ as well as decreasing mitochondrial ROS levels and caspase-3 activation in HL-1 mouse cardiomyocytes and in a mouse model of myocardial ischemia/reperfusion.^73^ Our findings are consistent with these reports and suggest that activation of SIRT1 and SIRT3 contributes to apoptosis suppression, forming part of the cardioprotective mechanism of dapagliflozin.

The metabolic alterations resulting from dapagliflozin administration may account for its observed capacity to activate sirtuin signaling and enhance mitochondrial performance. By promoting glucose excretion and a mild state of energy deficit, dapagliflozin activates crucial nutrient-sensing pathways, specifically 5′ adenosine monophosphate-activated protein kinase (AMPK) and SIRT1, which subsequently enhance mitochondrial biogenesis and optimize cellular energy utilization.^74,75^ Previous studies have shown that dapagliflozin upregulates SIRT1 and SIRT3 expression, enhances antioxidant capacity through GPX induction and reduces mitochondrial ROS generation, and inhibits apoptosis by increasing BCL-2 while decreasing BAX and caspase-3 in H9c2 cardiomyoblasts and rat myocardial infarction models,^29^ while SIRT3 activation similarly reduced postinfarction injury and oxidative stress in mice.^76^ Similarly, dapagliflozin has been shown to activate SIRT1-dependent pathways and enhance systemic energy metabolism.^77^ Collectively, systemic metabolic adaptations induced by dapagliflozin contribute to the enhanced cellular resistance observed in the present study.

Emerging evidence further indicates that dapagliflozin may exert direct myocardial effects through modulation of sodium-glucose cotransporters expressed in the heart.^30^ Although SGLT2 expression is minimal in healthy myocardium, it is upregulated under pathological conditions such as diabetes or heart failure, where dapagliflozin may inhibit abnormal glucose and sodium influx.^78^ Moreover, weak SGLT1 inhibition by dapagliflozin could reduce intracellular sodium and calcium overload, oxidative stress, and energetic inefficiency.^79^ Although our study did not directly investigate SGLT1 or SGLT2, these transporter-related impacts may represent an additional mechanism contributing to the overall cardioprotective profile of dapagliflozin. Furthermore, the potential clinical relevance of the current findings is significant for patients undergoing anthracycline chemotherapy. Cardiotoxicity remains one of the main limitations of doxorubicin therapy, and effective preventive strategies are limited. The results of this study indicate that dapagliflozin could provide cardiac protection without affecting the anticancer efficacy of chemotherapy. Previous clinical findings showed that treatment with dapagliflozin reduced doxorubicin-induced cardiac stress in patients and improved cardiac function without altering treatment outcomes.^32,80^ Additional clinical observations demonstrated that activation of stress response signaling contributed to the beneficial effects of dapagliflozin during doxorubicin exposure.^81,82^ These reports, together with the current findings, support further evaluation of dapagliflozin as an adjunctive cardioprotective agent during chemotherapy, targeting both metabolic and sirtuin-dependent myocardial pathways.

## 5 Conclusion

Dapagliflozin exerts strong cardioprotective potential against doxorubicin-induced cytotoxicity in H9c2 cardiomyoblasts by targeting multiple interrelated mechanisms (Figure 9). Dapagliflozin restores mitochondrial function, reduces oxidative stress, and suppresses both apoptotic and ferroptotic cell death. Central to these protective actions is the activation of the SIRT1/SIRT3 axis, which serves as a key upstream regulator of mitochondrial metabolism, redox homeostasis, and ferroptosis resistance. Notably, inhibition of either sirtuin abolished the protective effects of dapagliflozin, even when ferroptosis was pharmacologically blocked, underscoring the indispensable role of SIRT1/SIRT3 in mediating its efficacy. Pharmacological modulation of ferroptosis further underscores its contribution to doxorubicin cardiotoxicity and supports its targeting as a therapeutic strategy. In addition, our findings reveal a potential bidirectional crosstalk between sirtuin signaling and ferroptosis, suggesting that ferroptotic stress may, in turn, influence sirtuin expression. Collectively, this study highlights key mechanisms of dapagliflozin-mediated cardioprotection and supports the idea that modulating sirtuin and ferroptosis signaling may mitigate chemotherapy-induced cardiac damage. Further in vivo studies incorporating genetic validation, long-term outcome assessment, and clinical evaluation are required to confirm these findings and to establish the translational potential of dapagliflozin as a cardioprotective therapy during chemotherapy.

**Figure 9:**
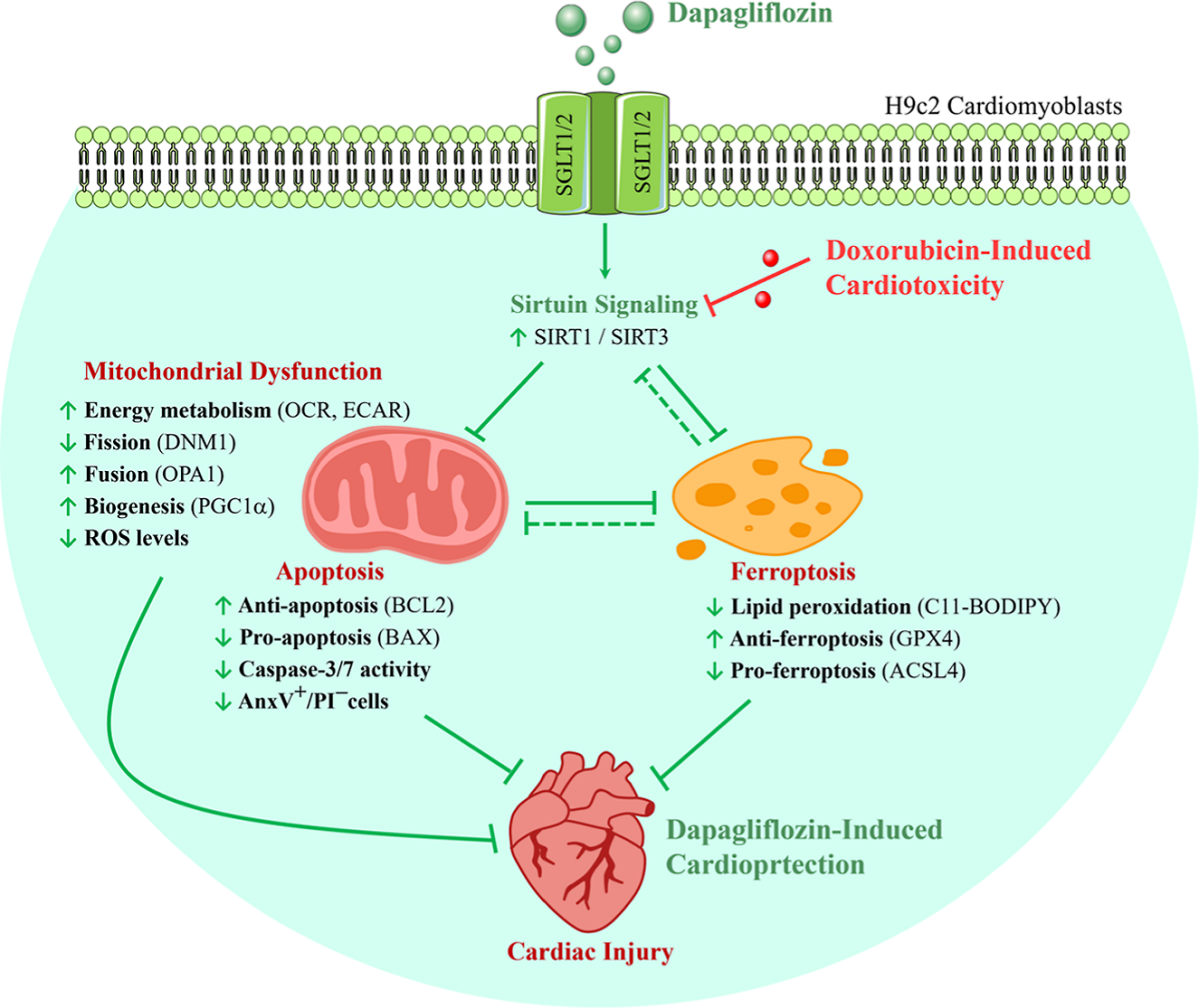
Cardioprotective mechanism of dapagliflozin in attenuating doxorubicin-induced cellular injuries through SIRT1/SIRT3 and ferroptosis regulation. Doxorubicin (Dox) induces cardiotoxicity by downregulating sirtuins, thereby promoting mitochondrial dysfunction, apoptosis, and ferroptosis, as evidenced by suppression of SIRT1/SIRT3, fusion (OPA1), biogenesis (PGC1α), antiapoptotic (BCL2), and antiferroptotic (GPX4) markers, along with activation of fission (DNM1), proapoptosis (BAX), and proferroptosis (ACSL4). Dapagliflozin (DAPA) mitigates Dox-induced cardiac injury through integrated activation of SIRT1/SIRT3 and inhibition of ferroptosis, ultimately restoring mitochondrial function, reducing oxidative and lipid damage, and enhancing cardiomyocyte survival. Interestingly, ferroptosis regulation reciprocally affects SIRT1/SIRT3 activity, suggesting potential feedback loops between sirtuin signaling and ferroptosis in DAPA-mediated cardiac defense. Green and red circles indicate gene up- and downregulation. Solid lines represent confirmed interactions, while dashed lines suggest potential feedback loops. Red arrows denote Dox-induced toxicity, and green arrows show DAPA-mediated cardioprotection.

## Supplementary Material



## Data Availability

All data generated or analyzed during this study are included in this published article.

## Notes

Figures S1–S4: The original, unprocessed Western blot images corresponding to those presented in the manuscript (PDF)

## Abbreviations

ACSL4: acyl-coenzyme A synthetase long-chain family member 4
ATP: adenosine triphosphate
BAX: B-cell lymphoma 2-associated X protein
BCL2: B-cell lymphoma 2
DNM1: dynamin 1
ECAR: extracellular acidification rate
GPX4: glutathione peroxidase 4
NAD^+^: nicotinamide adenine dinucleotide
OCR: oxygen consumption rate
OPA1: optic atrophy 1
PGC-1α: peroxisome proliferator-activated receptor gamma coactivator 1-alpha
p53: tumor protein p53
ROS: reactive oxygen species
SGLT2: sodium-glucose cotransporter 2
SIRT1: sirtuin 1
SIRT3: sirtuin 3
