# Supplementary material for: Dapagliflozin Protects Cardiomyocytes against Doxorubicin-Induced Toxicity by Modulating Sirtuin 1/Sirtuin 3 and Ferroptosis Pathway
Source: ACS Pharmacol Transl Sci. 2026 Apr 3;9(5):1134–52. doi: 10.1021/acsptsci.5c00760 (PMC13162165; doi:10.1021/acsptsci.5c00760)

SUPPORTING INFORMATION

Dapagliflozin protects cardiomyocytes against doxorubicin-induced toxicity by modulating sirtuin 1/sirtuin 3 and ferroptosis pathway

Hnin Ei Ei Khine[1], *Supachoke Mangmool*[2], *Warisara Parichatikanond*[1,3,4,*]

[1] Department of Pharmacology, Faculty of Pharmacy, Mahidol University, Bangkok 10400, Thailand

[2] Department of Pharmaceutical Care, Faculty of Pharmacy, Chiang Mai University, Chiang Mai 50200, Thailand

[3] Center of Biopharmaceutical Science for Healthy Ageing, Faculty of Pharmacy, Mahidol University, Bangkok 10400, Thailand

[4] Centre of Molecular Targeting and Integrated Drug Development, Faculty of Pharmacy, Mahidol University, Bangkok 10400, Thailand

*** Corresponding author:**

Warisara Parichatikanond, Ph.D.

Department of Pharmacology, Faculty of Pharmacy, Mahidol University

447 Sri-Ayuthaya Road, Rajathevi, Bangkok 10400 Thailand

Phone: +66-922464498, E-mail: warisara.par@mahidol.ac.th

Table of Contents for Supporting Information

Figure S1. Original western blot images supporting **Figure 2**, showing that dapagliflozin alleviates doxorubicin-induced cardiotoxicity in H9c2 cells.. **S-3**

Figure S2. Original western blot images supporting **Figure 4**, indicating that inhibition of SIRT1 or SIRT3 reduces the protective effects of dapagliflozin in doxorubicin-exposed H9c2 cells............ **S-4**

Figure S3. Original western blot images supporting **Figure 6**, demonstrating that modulation of ferroptosis influences the protective response to dapagliflozin during doxorubicin challenge in H9c2 cells... **S-5**

Figure S4. Original western blot images supporting **Figure 8**, revealing that the protective effects of dapagliflozin under ferroptosis inhibition require functional SIRT1 and SIRT3 in doxorubicin-treated H9c2 cells... **S-6**

Figure S1. Original western blot images supporting **Figure 2**, showing that dapagliflozin alleviates doxorubicin-induced cardiotoxicity in H9c2 cells.

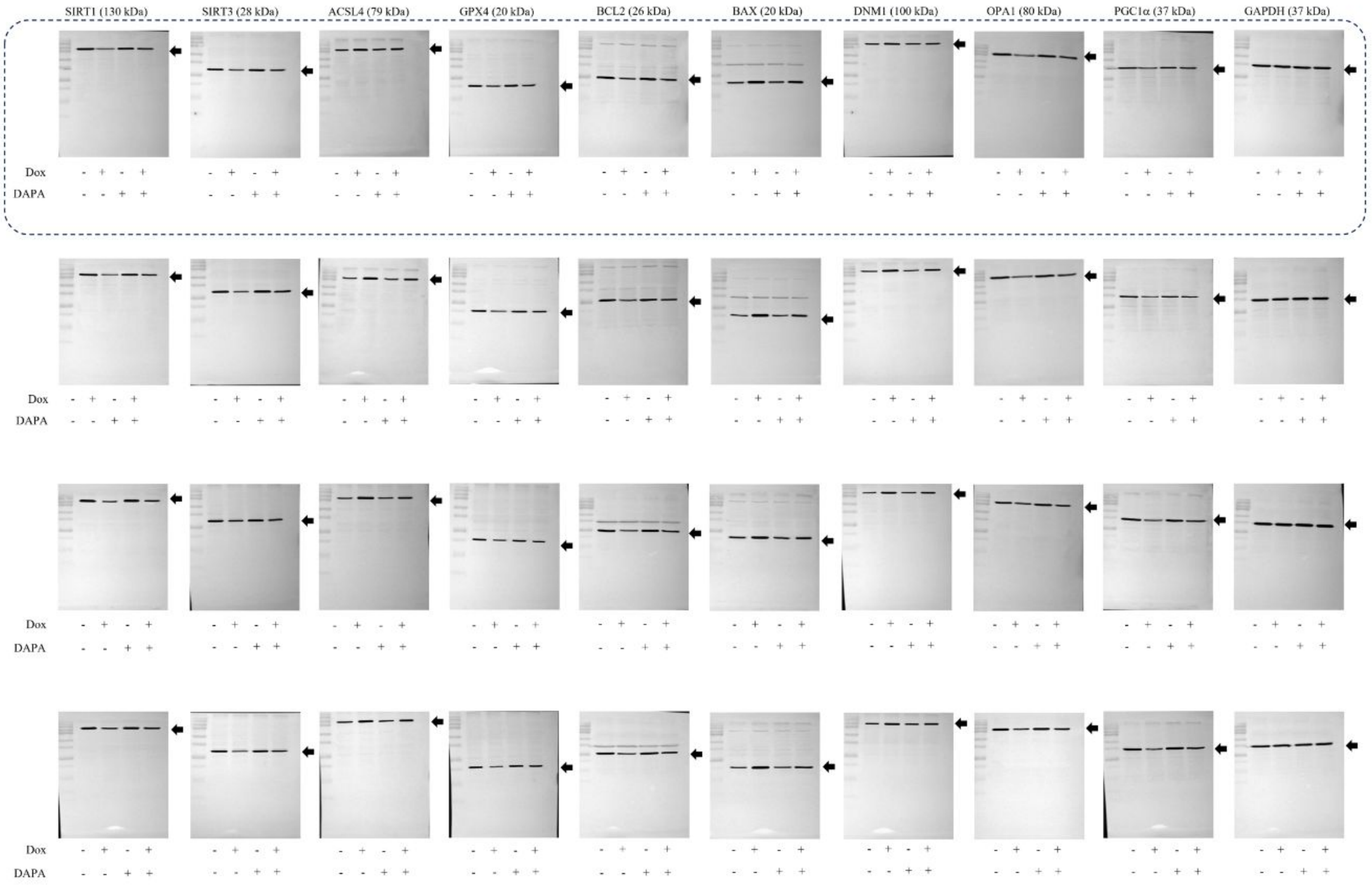

Figure S2. Original western blot images supporting **Figure 4**, indicating that inhibition of SIRT1 or SIRT3 reduces the protective effects of dapagliflozin in doxorubicin-exposed H9c2 cells.

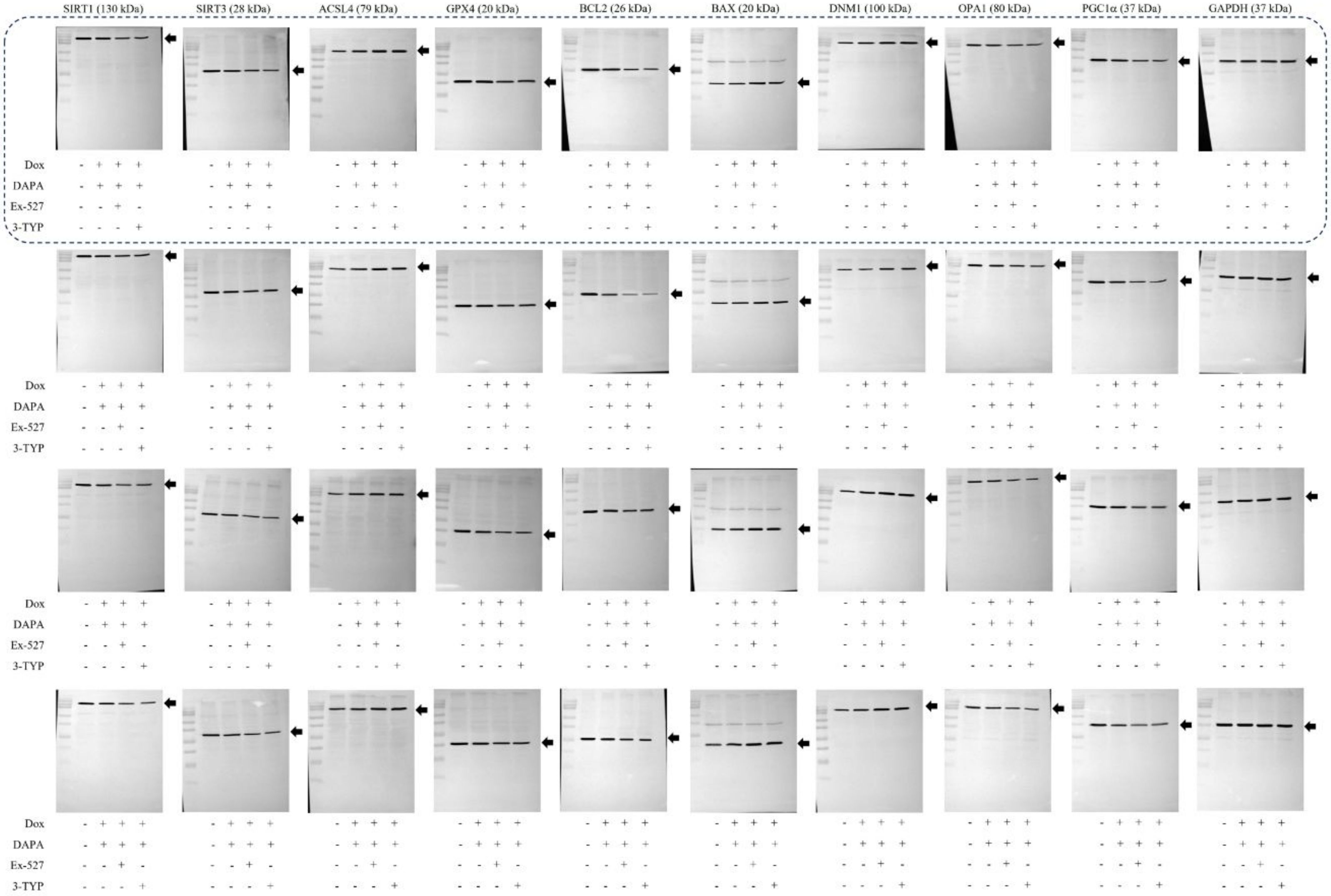

Figure S3. Original western blot images supporting **Figure 6**, demonstrating that modulation of ferroptosis influences the protective response to dapagliflozin during doxorubicin challenge in H9c2 cells.

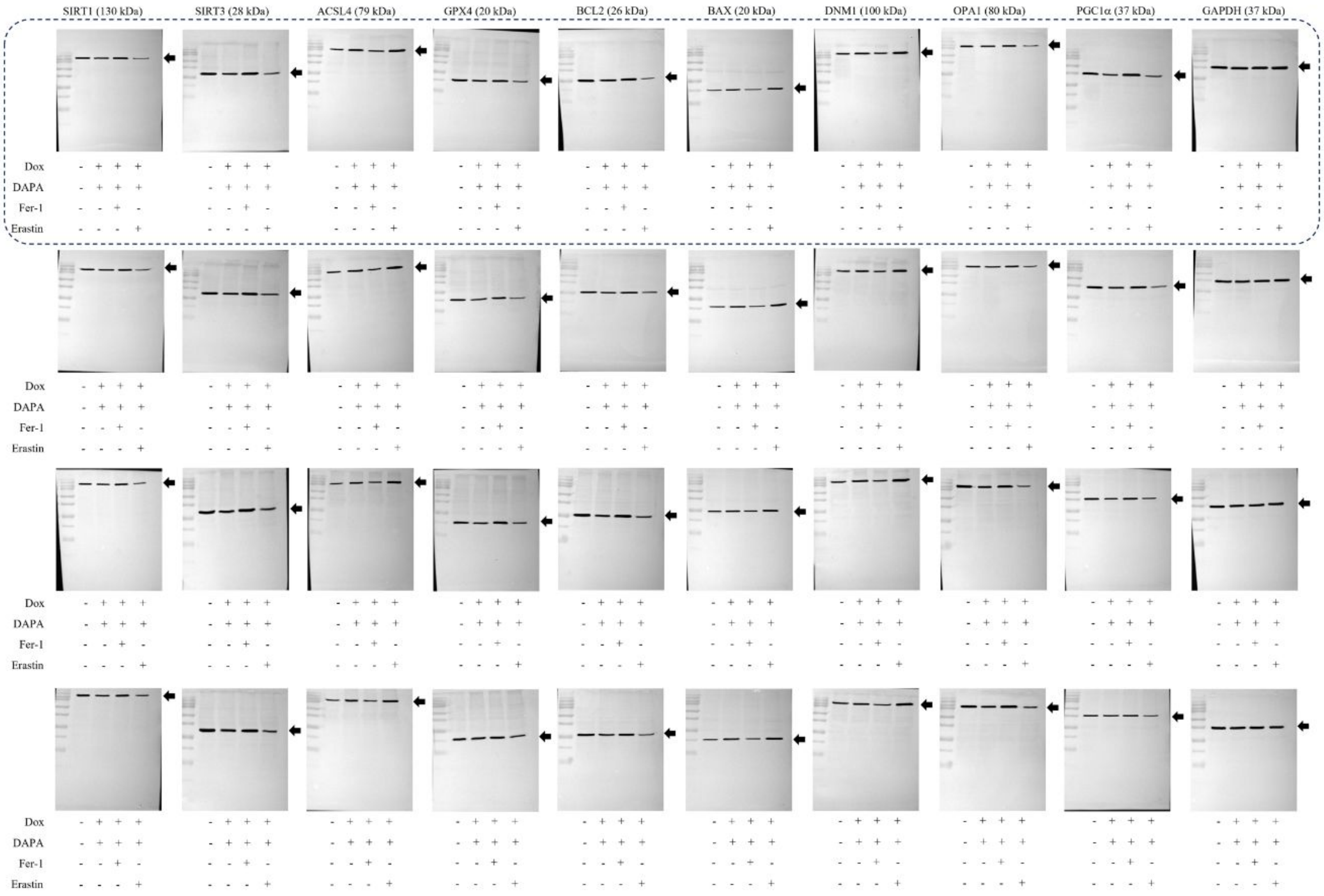

Figure S4. Original western blot images supporting **Figure 8**, revealing that the protective effects of dapagliflozin under ferroptosis inhibition require functional SIRT1 and SIRT3 in doxorubicin-treated H9c2 cells.

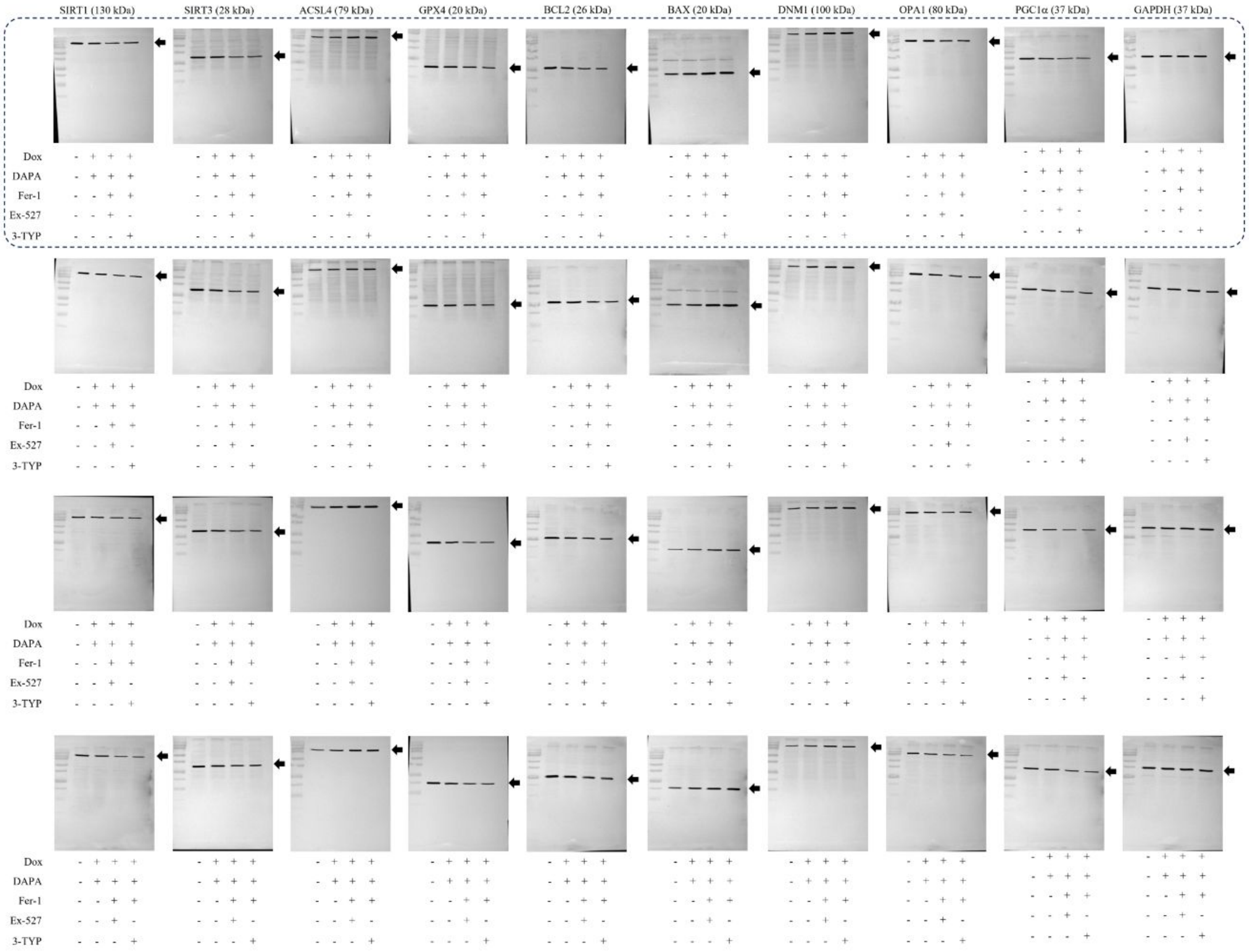

Supplement: Supplementary file 1 [file pt5c00760_si_001.pdf]
